# Provenance Information for Biomedical Data and Workflows: Scoping Review

**DOI:** 10.2196/51297

**Published:** 2024-08-23

**Authors:** Kerstin Gierend, Frank Krüger, Sascha Genehr, Francisca Hartmann, Fabian Siegel, Dagmar Waltemath, Thomas Ganslandt, Atinkut Alamirrew Zeleke

**Affiliations:** 1 Department of Biomedical Informatics Mannheim Institute for intelligent Systems in Medicine Medical Faculty Mannheim, Heidelberg University Mannheim Germany; 2 Faculty of Engineering Wismar University of Applied Sciences Wismar Germany; 3 Institute of Communications Engineering University of Rostock Rostock Germany; 4 Department of Medical Informatics University Medicine Greifswald Greifswald Germany; 5 Chair of Medical Informatics Friedrich-Alexander-Universität Erlangen-Nürnberg Erlangen Germany

**Keywords:** provenance, biomedical research, data management, scoping review, health care data, software life cycle

## Abstract

**Background:**

The record of the origin and the history of data, known as provenance, holds importance. Provenance information leads to higher interpretability of scientific results and enables reliable collaboration and data sharing. However, the lack of comprehensive evidence on provenance approaches hinders the uptake of good scientific practice in clinical research.

**Objective:**

This scoping review aims to identify approaches and criteria for provenance tracking in the biomedical domain. We reviewed the state-of-the-art frameworks, associated artifacts, and methodologies for provenance tracking.

**Methods:**

This scoping review followed the methodological framework developed by Arksey and O’Malley. We searched the PubMed and Web of Science databases for English-language articles published from 2006 to 2022. Title and abstract screening were carried out by 4 independent reviewers using the Rayyan screening tool. A majority vote was required for consent on the eligibility of papers based on the defined inclusion and exclusion criteria. Full-text reading and screening were performed independently by 2 reviewers, and information was extracted into a pretested template for the 5 research questions. Disagreements were resolved by a domain expert. The study protocol has previously been published.

**Results:**

The search resulted in a total of 764 papers. Of 624 identified, deduplicated papers, 66 (10.6%) studies fulfilled the inclusion criteria. We identified diverse provenance-tracking approaches ranging from practical provenance processing and managing to theoretical frameworks distinguishing diverse concepts and details of data and metadata models, provenance components, and notations. A substantial majority investigated underlying requirements to varying extents and validation intensities but lacked completeness in provenance coverage. Mostly, cited requirements concerned the knowledge about data integrity and reproducibility. Moreover, these revolved around robust data quality assessments, consistent policies for sensitive data protection, improved user interfaces, and automated ontology development. We found that different stakeholder groups benefit from the availability of provenance information. Thereby, we recognized that the term *provenance* is subjected to an evolutionary and technical process with multifaceted meanings and roles. Challenges included organizational and technical issues linked to data annotation, provenance modeling, and performance, amplified by subsequent matters such as enhanced provenance information and quality principles.

**Conclusions:**

As data volumes grow and computing power increases, the challenge of scaling provenance systems to handle data efficiently and assist complex queries intensifies, necessitating automated and scalable solutions. With rising legal and scientific demands, there is an urgent need for greater transparency in implementing provenance systems in research projects, despite the challenges of unresolved granularity and knowledge bottlenecks. We believe that our recommendations enable quality and guide the implementation of auditable and measurable provenance approaches as well as solutions in the daily tasks of biomedical scientists.

**International Registered Report Identifier (IRRID):**

RR2-10.2196/31750

## Introduction

### Background

Both the use and reuse of electronic medical and patient-related data offers enormous potential for clinical research [1,2]. National programs such as the German Medical Informatics Initiatives support knowledge discovery and data sharing using adequate computational infrastructure and secure processes [3]. In this context, provenance information (Textbox 1) offers access to quality assured, traceable, and credible shared data. These and other advantages of data provenance have been demonstrated, for instance, in the EU Horizon 2020 TRANSFORM project [4] or in the MeDaX Knowledge Graph Prototype [5]. Schröder et al [6] provided an “Electronic Laboratory Notebook” use case in the wet lab to show how provenance supports the understanding and reproducibility of research investigations. At the same time, researchers not considering the origin of data run into the hazard of systematically incomplete or wrong data [7].

Notably, the FAIR (findable, accessible, interoperable, reusable) guiding principles for data stewardship [9] explicitly mention provenance [10,11]. A provenance-oriented approach requires thorough planning, execution, and evaluation of data management processes in the respective application domain [2]. In the scientific context, adherence to criteria such as consistency, interoperability, and confidentiality are generally required across all software tools [1,12,13].

The concept and implementation of provenance are essential for most scientific domains, such as environmental fields (geoprocessing workflows or climate assessments), in nuclear fusion engineering, or material sciences [14,15]. In particular, the biomedical domains demand comprehensive investigation and information about their data management scenarios, including extract, transform, load jobs for data transfer and integration. Reliable data and data pipelines both require provenance data to be embedded in concepts for traceability to understand the relationships between results and source data.

**Provenance terminology.**

**Definition**

- “Provenance” is a description of what happened to a data item [4]. Information models such as the World Wide Web Consortium (W3C) PROV standard formally define provenance of a resource as “a record that describes entities and processes involved in producing and delivering or otherwise influencing that resource” [8].

### Objectives

Our work reviews approaches and criteria for provenance tracking in the biomedical domain and discloses current knowledge gaps. This comprises modeling aspects and metadata frameworks for meaningful and usable provenance information during the creation, collection, and processing of scientific biomedical data. The review also covers the examination of quality aspects relating to provenance.

## Methods

### Overview

We followed the scoping methodological framework developed by Arksey and O’Malley [16] for conducting a scoping review with the following stages: (1) stage 1—identification of the research questions (RQs); (2) stage 2—identification of relevant studies; (3) stage 3—study selection; (4) stage 4—data extraction and charting; and (5) stage 5—collating, summarizing, and reporting the results.

### Change From the Original Protocol

The protocol of this scoping review has been published in *JMIR Research Protocols* (international registered report identifier DERR1-10.2196/31750) [17]. In accordance with the original protocol developed for this scoping review, the search period was initially planned to include studies published from January 2006 to March 2021. However, due to the extensive nature of the data extraction and write-up process, it became apparent that additional time was necessary to ensure a comprehensive analysis of the relevant literature. As a result, we extended the search period from 2006 to the end of 2022.

No other changes were made to the original protocol. Thematic analysis methods were applied to analyze the extracted data by organizing themes according to the RQs [18]. In line with the framework developed by Arksey and O’Malley [16], the review does not attempt to assess the quality of studies or the risk of bias. It also does not assess the generalizability of the results.

### Stage 1: Identifying RQs

The main objective of this review was to investigate existing evidence regarding approaches and criteria for provenance tracking and disclosing current knowledge gaps in the biomedical domain. The objective led to the following RQs:

- RQ1: Which potential (methodological) approaches exist for the classification and tracking of provenance criteria and methods in a biomedical or domain-independent context?
- RQ2: How can the potential value of provenance information be harnessed and by whom? How can usability be provided?
- RQ3: What are the challenges and potential problems or bottlenecks for the accomplishment of provenance?
- RQ4: Which guidelines or demands for the consideration of provenance criteria in a biomedical or domain-independent context have to be followed?
- RQ5: How completely can provenance be mapped in the data life cycle or during data management?

### Stage 2: Identifying Relevant Studies

Concepts and matching keywords were categorized into 4 groups (Table 1): *target domain* refers to the context of the research topic and includes studies with a biomedical, health care, clinical, or scientific background. In this work, scientific background is limited to domain-independent studies and excludes all other domain-specific studies. *Provenance* concerns the information about the genesis of a given object. *Provenance properties* cover specific requirements tied to the term *provenance*; they also describe selected characteristics in this context. *Objective* includes the purposes or intention of provenance capture. The comprehensive search strategy is recorded in the study protocol [17], and search strings combined with Boolean operators are attached (Multimedia Appendix 1).

**Table 1:** Concepts and matching keywords (eligibility criteria).

Concepts	Matching keywords (inclusion criteria)
Target domain	biomed*^a^, EHR, electronic health record, health care, clinical, scientific^b^
Provenance	provenance, prov, lineage
Provenance properties	interop*, (data NEAR/2 [flow, quality, transformation]), metadata, workflow, semantic, framework, annotat*, ontolog*, management, document*, (model NEAR/2 provenance)
Objective	audit*, decision support, ETL, Extract-Transform-Load, FHIR, record linking, machine learning, reproducib*, transparen*, track*, implement*

^a^The * symbol (wildcard character) replaces or represents one or more characters.

^b^Will be used in a domain-independent context only.

### Stage 3: Study Selection

The PRISMA (Preferred Reporting Items for Systematic Reviews and Meta-Analyses) flowchart depicts the selection process. First, we identified all relevant studies in the PubMed and Web of Science databases based on our search strategy. After deduplication, we launched a transparent screening process by importing all relevant studies into Rayyan [19], a systematic review supporting solution. The studies were then reviewed by at least 2 independent researchers. In the case of vote agreement, the study was either included in the next review phase or excluded from the review. A third independent reviewer was consulted to solve the conflict if no consensus could be reached. The study screening phase started with a title and abstract evaluation for eligibility. Included studies were submitted to a full-text screening, while performing a thorough investigation on the study report. Reviewers voted for inclusion or exclusion considering the inclusion and exclusion criteria. Finally, the residing set of qualified studies was moved into the data extraction pipeline. A description of the study selection is provided in the protocol [17].

Studies were included if they (1) were focused on the biomedical domain or were domain independent, (2) described provenance-tracking approaches, and (3) were written in English. Studies were excluded if they (1) were not specific to the biomedical or general domain, (2) were gray literature, and (3) did not focus on provenance-tracking approaches.

### Stage 4: Charting the Data

We followed a collaborative and iterative process to define a charting table for data extraction. Individual reviewers (KG, FK, FH, SG, AAZ, and DW) then scrutinized all studies and extracted central textual occurrences into the data extraction sheet. The variables in the data extraction sheet correspond with the RQs. General characteristics of the studies, approaches for classification and tracking of provenance, and their associated challenges along with the significance and completeness of provenance information in the given context were part of the investigational charting. The reviewers independently charted the data in a structured and consistent way and discussed the results.

### Stage 5: Collating, Summarizing, and Reporting the Results

The extracted data were analyzed using summary statistics by calculating the total number and percentages of all studies per category, if applicable. Charts were presented for the distribution of the individual data elements where applicable. The data analysis was partially supported with scripts in Python (version 3.10.0) [20]. Plots were generated with R version 4.0.4 (R Core Team) [21] and version 1.3.0 of the *tidyverse* package [22].

Further analysis was performed using qualitative evaluation. The reporting of the results and outcomes was structured according to the RQs. On the basis of the analysis of the review results, we have developed a road map for a customized provenance framework that considers the life cycle of the software framework (Provenance-Software Framework Life Cycle [SFL]). Implications for future research, practice, and policy makers were outlined. Our reporting adheres to the PRISMA-ScR (Preferred Reporting Items for Systematic Reviews and Meta-Analyses Extension for Scoping Reviews) reporting guidelines [23].

## Results

### Literature Search

**Figure 1:**
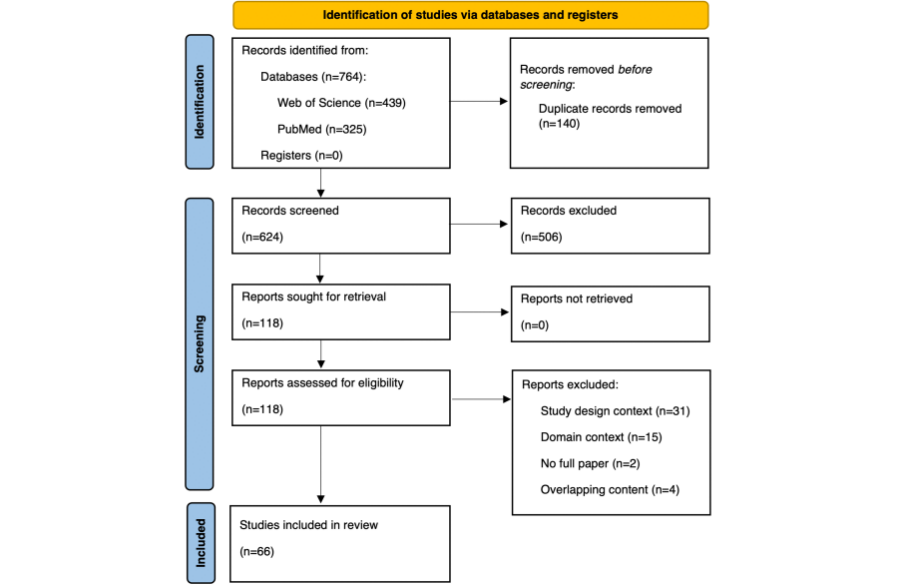
PRISMA (Preferred Reporting Items for Systematic Reviews and Meta-Analyses) flow diagram displaying the paper selection process with the number of studies in the identification and screening phases and all included studies in the scoping review.

The search in the PubMed and Web of Science databases resulted in 764 hits and included papers from January 1, 2006, to December 31, 2022. Afterward, 140 duplicates were removed. The remaining 624 papers were subjected to title-abstract screening in an interactive selection process, leaving 118 eligible papers for the full-text review. The full-text papers were further screened to identify papers eligible for the subsequent step of data charting. During this step, additional 52 papers were excluded (see the Stage 4: Charting the Data section). These papers either did not meet the study design context (31/52, 60%) or they lacked the domain concept (15/52, 29%). Four papers reported the same study or contained parts of it, and 2 were not a full paper. A total of 66 articles were included in the data extraction phase (Table 2). The paper selection followed the PRISMA [24] approach (Figure 1), and the PRISMA-ScR checklist is presented in Multimedia Appendix 2.

**Table 2:** List of included papers.

Number	Title	Study
1	Clinical Text Mining on FHIR	Daumke et al [25], 2019
2	Provenance Solutions for Medical Research in Heterogeneous IT-Infrastructure: An Implementation Roadmap	Parciak et al [26], 2019
3	BioWorkbench: a high-performance framework for managing and analyzing bioinformatics experiments	Mondelli et al [27], 2018
4	Towards structured sharing of raw and derived neuroimaging data across existing resources	Keator et al [28], 2013
5	A unified framework for managing provenance information in translational research	Sahoo et al [13], 2011
6	Towards FAIR protocols and workflows: the OpenPREDICT use case	Celebi et al [29], 2020
7	A Survey on Collecting, Managing, and Analyzing Provenance from Scripts	Pimentel et al [30], 2019
8	Reproducibility Analysis of Scientific Workflows	Bánáti et al [31], 2017
9	Implementing interoperable provenance in biomedical research	Curcin et al [1], 2014
10	Why linked data is not enough for scientists	Bechhofer et al [32], 2013
11	Representing distributed systems using the Open Provenance Model	Groth and Moreau [33], 2011
12	A Semantic Web approach to the provenance challenge	Golbeck and Hendler [34], 2008
13	Applying content management to automated provenance capture	Schuchardt et al [35], 2008
14	The Generalized Data Model for clinical research	Danese et al [36], 2019
15	FHIR Healthcare Directories: Adopting Shared Interfaces to Achieve Interoperable Medical Device Data Integration	Tyndall and Tyndall [37], 2018
16	ProvCaRe: Characterizing Scientific Reproducibility of Biomedical Research Studies using Semantic Provenance Metadata	Sahoo et al [38], 2019
17	Embedding data provenance into the Learning Health System to facilitate reproducible research	Curcin [4], 2017
18	Application of Data Provenance in Healthcare Analytics Software Information Visualisation of User Activities	Xu et al [39], 2018
19	AiiDA 1.0, a scalable computational infrastructure for automated reproducible workflows and data provenance	Huber et al [40], 2020
20	Provenance for distributed biomedical workflow execution	Madougou et al [41], 2012
21	Capturing and Analyzing Provenance from Spark-based Scientific Workflows with SAMbA-RaP	Guedes et al [42], 2020
22	Why-Diff: Exploiting Provenance to Understand Outcome Differences From Non-Identical Reproduced Workflows	Thavasimani et al [43], 2019
23	Deriving scientific workflows from algebraic experiment lines: A practical approach	Marinho et al [44], 2017
24	Access control and view generation for provenance graphs	Danger et al [45], 2015
25	Provenance-based reproducibility in the Semantic Web	Moreau [46], 2011
26	PASSing the Provenance challenge	Holland et al [47], 2008
27	Decentralised provenance for healthcare data	Margheri et al [48], 2020
28	Enhancing Traceability in Clinical Research Data through a Metadata Framework.	Hume et al [12], 2020
29	Scientific Reproducibility in Biomedical Research: Provenance Metadata Ontology for Semantic Annotation of Study Description	Sahoo et al [49], 2016
30	Managing and exploiting routinely collected NHS data for research	Curcin et al [50], 2013
31	The eGenVar data management system—cataloguing and sharing sensitive data and metadata for the life sciences	Razick et al [51], 2014
32	BioQ: tracing experimental origins in public genomic databases using a novel data provenance model	Saccone et al [52], 2012
33	Applications of provenance in performance prediction and data storage optimisation	Woodman et al [53], 2017
34	ProvManager: a provenance management system for scientific workflows	Marinho et al [54], 2012
35	Provenance in collection-oriented scientific workflows	Bowers et al [55], 2008
36	A novel approach to provenance management for privacy preservation	Can and Yilmazer [56], 2020
37	Templates as a method for implementing data provenance in decision support systems	Curcin et al [57], 2017
38	Sharing interoperable workflow provenance: A review of best practices and their practical application in CWLProv	Khan et al [58], 2019
39	PAV ontology: provenance, authoring and versioning	Ciccarese et al [59], 2013
40	Enabling precision medicine via standard communication of HTS provenance, analysis, and results	Alterovitz et al [60], 2018
41	NeuroProv: Provenance data visualisation for neuroimaging analyses	Arshad et al [61], 2019
42	AVOCADO: Visualization of Workflow-Derived Data Provenance for Reproducible Biomedical Research	Stitz et al [62], 2016
43	Provenance Context Entity (PaCE): Scalable Provenance Tracking for Scientific RDF Data	Sahoo et al [63], 2010
44	A semantic proteomics dashboard (SemPoD) for data management in translational research	Jayapandian et al [2], 2012
45	Providing traceability for neuroimaging analyses	McClatchey et al [64], 2013
46	PGxO and PGxLOD: a reconciliation of pharmacogenomic knowledge of various provenances, enabling further comparison	Monnin et al [65], 2019
47	Provenance data discovery through Semantic Web resources	Ornelas et al [66], 2018
48	Characterizing workflow-based activity on a production e-infrastructure using provenance data	Madougou et al [67], 2013
49	Storing, reasoning, and querying OPM-compliant scientific workflow provenance using relational databases	Lim et al [68], 2011
50	Blockchain for Healthcare: Securing Patient Data and Enabling Trusted Artiﬁcial Intelligence	Jennath et al [69], 2020
51	Bio-Swarm-Pipeline: a light-weight, extensible batch processing system for efficient biomedical data processing	Cheng et al [70], 2009
52	A Comprehensive Query Language for Provenance Information	Jabal and Bertino [71], 2018
53	Provenance trails in the Wings/Pegasus system	Kim et al [72], 2008
54	OPQL: Querying scientific workflow provenance at the graph level	Lim et al [73], 2013
55	Lightweight Distributed Provenance Model for Complex Real-world Environments	Wittner et al [74], 2022
56	A collaborative semantic-based provenance management platform for reproducibility	Samuel and Konig-Ries [75], 2022
57	A novel visualization approach for data provenance	Yazici et al [76], 2022
58	Trellis for efficient data and task management in the VA Million Veteran Program	Ross et al [77], 2021
59	ECO: the Evidence and Conclusion Ontology, an update for 2022	Nadendla et al [78], 2022
60	RepeatFS: a file system providing reproducibility through provenance and automation.	Westbrook et al [79], 2021
61	The BMS-LM ontology for biomedical data reporting throughout the life cycle of a research study: From data model to ontology	Raboudi et al [80], 2022
62	FAIRSCAPE: a Framework for FAIR and Reproducible Biomedical Analytics	Levinson et al [81], 2021
63	FAIRly big: A framework for computationally reproducible processing of large-scale data	Wagner et al [82], 2022
64	FAIR data pipeline: provenance-driven data management for traceable scientific workflows	Mitchell et al [83], 2022
65	Enabling Scientific Reproducibility through FAIR Data Management: An ontology-driven deep learning approach in the NeuroBridge Project	Wang et al [84], 2022
66	The Neuroscience Experiments System (NES)-A Software Tool to Manage Experimental Data and Its Provenance	Ruiz-Olazar et al [85], 2021

### Characteristics of the Included Studies

All papers (n=66) were first published between 2006 and 2022 (Table 2). More than half of the reviewed studies were published within the past 5 years, which we selected for our scoping review. Predominantly, studies originated from the biomedical or health care domain (45/66, 68%), followed by the domain-independent studies (21/66, 32%). Document characteristics of the study corpus are provided in Multimedia Appendix 3.

All studies in this review were screened with regard to the 5 RQs described in the Methods section. The following subsections describe our findings for RQ1 to RQ5.

### RQ1: Classification and Tracking of Provenance Criteria in Biomedical Workflows and Data

#### RQ1.1: Characteristics of Provenance Framework Types

Heterogeneous approaches for classifying and tracking provenance criteria have been reported in the selected literature (n=66). We propose to categorize them by their focus (Multimedia Appendix 4). Most articles (58/66, 88%) focused explicitly on practical provenance management approaches. The remaining theoretical frameworks (8/66, 12%) provided recommendations or reviews. They can be classified into the following subcategories.

#### Semantics and Models, Ontologies, and Metadata (27/58, 47%)

This comprises provenance-tracking approaches on different granularity, ontology, and model abstraction levels. The semantic Provenance Context Entity approach [63] was developed to track provenance in Resource Description Framework–based semantic web applications. An example of an annotation mechanism was introduced with collection-oriented modeling and design [55]. The Provenance Metadata Model (ProvCaRe S3), supporting scientific reproducibility, was represented with the Web Ontology Language and provenance triples served as a basis for the provenance graph [38]. Later, the NeuroBridge ontology extended ProvCaRe, combined with a deep learning model [84]. Further ontologies include the REPRODUCE-ME ontology, integrated in the CAESAR project [75], or the BioMedical Study–Lifecycle Management core ontology [80]. application programming interfaces for visualization [71] or querying purposes [28] and web services for user access to provenance data [2] were reported.

#### Scientific Workflows and Workflow Executions (18/58, 31%)

These are mainly Open Provenance Model (OPM)–oriented workflows [86] on different semantic levels, like in the BioWorkbench [27], OpenPREDICT [29], or Web Ontology Language projects. Provenance data were stored in relational databases, like in OPMProv [73] or in graph databases [53,77]. Querying possibilities were offered via a web service or with specific querying languages at the graph level [73].

#### Privacy Aspects (5/58, 9%)

Decentralized management and General Data Protection Regulation requirements led to the use of blockchain technologies [48] in combination with the PROV model standard. Another scenario incorporated blockchain in a proof-of-concept study [69] to enable an audit trail mechanism for a trusted artificial intelligence model.

#### Visualization Aspect (4/58, 7%)

The complexity of representing provenance information at different levels of aggregation was examined in the AVOCADO project [62]. The NeuroProv project [61] shows how visualization supports clinicians in information tracking and reproducibility analysis.

#### General Data Managing Tools (4/58, 7%)

Frameworks provide different modules for data and workflow provenance capture, representation, storage, comparison, and visualization [75] or automatic recomputation of arbitrary data-processing results [82].

#### RQ1.2: Provenance Model Characteristics

At all, 58 papers reported about provenance model characteristics. The dominant provenance models refer to the PROV [8] specification (25/58, 43%), established by the World Wide Web Consortium (W3C) as the de facto standard for provenance modeling, and the frequently used OPM [86] (17/58, 29%; Multimedia Appendix 5). Other models cite specific solutions (11/58, 19%), are concerned with metadata provision (5/58, 9%), or do not provide any information on the provenance model (8/58, 14%).

OPM is the result of 3 provenance challenges (since 2011 until today). OPM (version 1.1) is exchangeable across systems and supports a process-oriented and dataflow-oriented view. It is based on the notion of the annotated causality graph with nodes as artifacts, processes, and agents. OPM was further developed into a provenance data model. PROV [8] comprises a family of specifications for provenance, designed to promote the publication of provenance information on the web. It offers interoperability across systems and is quite generic.

**Figure 2:**
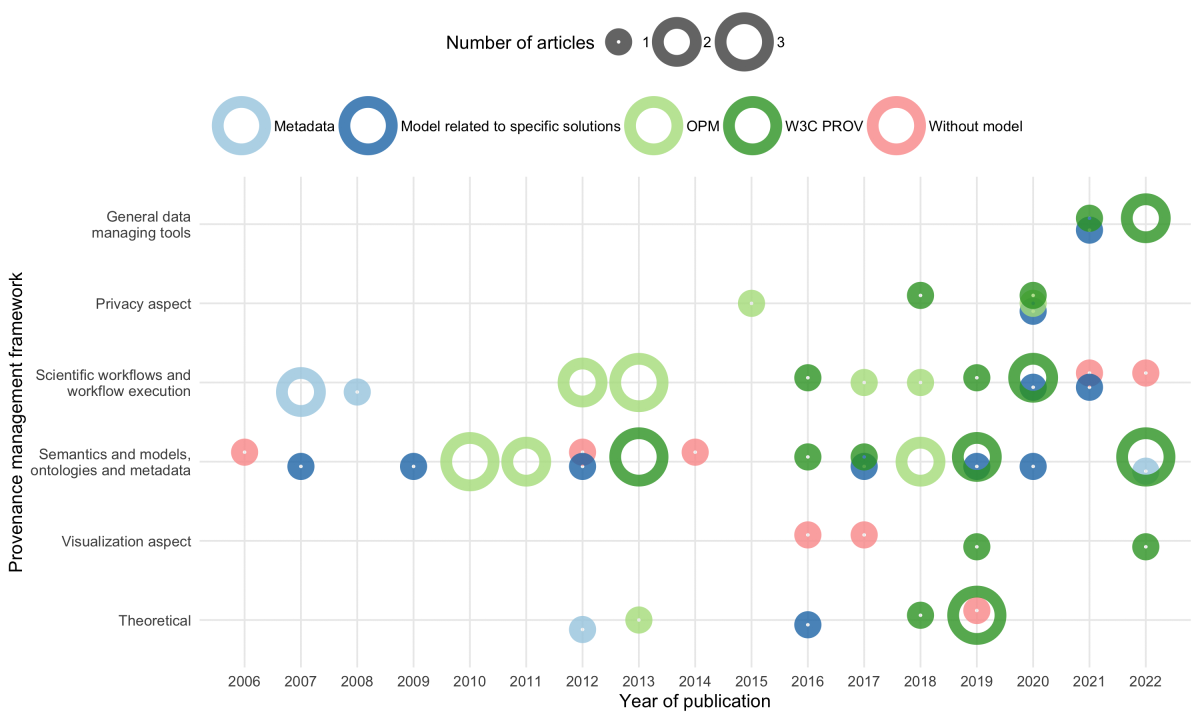
Reported provenance management frameworks per year. The size of the ring corresponds with the number of articles per year that discuss a specific model (color-coded) in the context of the respective framework. OPM: Open Provenance Model; W3C: World Wide Web Consortium.

Figure 2 displays the temporal evolution of the characterized frameworks depending on the applied models. We observed an increased number of papers relating to these provenance management frameworks between 2016 and 2022. At this time, the OPM and W3C PROV standards were extended. The onset of the FAIR principles [29] and the Fast Healthcare Interoperability Resources framework [40] furthermore set new requirements for modeling and implementation projects.

#### RQ1.3: Validation Status

Most of the studies (n=52) report a successful validation of their provenance solution. The use cases were mostly domain-specific (eg, as part of the AVOCADO [62] project). Other authors chose classical semantic evaluation schemes that demonstrated feasibility by responding to competency questions. Examples are the provenance challenges or proof-of-concept frameworks [13,29,56,69,72].

To pass the provenance challenges, participants needed to solve predefined provenance queries [47,55,73]. Can and Yilmazer [56] evaluated their domain-independent model with an infectious disease use case and implementing the Healthcare Provenance Information System. Sometimes, more than one approach had been taken for evaluation (eg, use cases and factors, ontology validation, and a user-based study were applied to evaluate the CAESAR approach for scientific experiments [75]). A prototype visualization tool for provenance information used satisfaction surveys to assess the usability of the system [76].

Curcin et al [57] emphasized that the setup of provenance data needs to be modeled and verified separately from the software implementation. Precise validation methods for provenance services focus on usability, performance, scalability, fault tolerance, and functionality [64]. Moreover, they demanded more formal engineering techniques to foster provenance implementation across a broad range of software tools in the biomedical domain and beyond [1,74]. In that sense, formal validation as part of the software engineering process contributes to increased software quality, and formal validation requires testing efforts and testing evidence. However, accurate alignment of testing procedures against predefined requirements in the software life cycle could not be identified.

#### RQ1.4: Provenance Characteristics

The term *provenance* is subjected to an evolutionary and technical process with multifaceted meanings and roles in the selected papers (n=66). There is agreement that provenance is a piece of history. However, the focus of provenance work ranges from abstract workflow descriptions to summaries of workflow executions to more general knowledge about data sources and result dependencies [2,51,65,72,73]. For example, provenance as semantic metadata was specified in several works between 2007 and 2019. Monnin et al [65] required the encoding of provenance of pharmacogenomics knowledge units. Other works refer to data provenance as knowledge about data sources [45] or as a piece of analytic software [39], as machine-interpretable provenance of data sets, as software and computations, as metadata for all computed results [81], or as the description of the data and its original context, and tracing data history from their creation to their sharing [80].

Sahoo et al [38] stated that the provenance data model together with the PROV Ontology define the minimal categories of provenance metadata terms. Other studies discussed the combined provenance of data and workflows and introduce the terms *prospective*, *retrospective*, and *domain provenance* [1,42,59]. While prospective provenance expresses future abstract workflow information, retrospective provenance gathers past workflow execution and data derivation information. Domain-specific provenance can be defined as an extension to the PROV Ontology. Workflow provenance has repeatedly been mentioned in the context of workflow execution [27,31,34]. Wittner et al [74] introduced the term *provenance backbone*, which covers coarse granularity representation of traceable object artifacts, whereas Mitchell et al [83] expressed provenance in relation to intrinsic and extrinsic metadata. The “FAIRly big framework” [82] demonstrates how records of process provenance are captured and stored in a machine-readable, automatically re-executable way. The 7 Ws (who, what, where, why, when, which, and how) characterize provenance in the study by Ruiz-Olazar et al [85].

#### RQ1.5: Requirements for Provenance Frameworks

**Figure 3:**
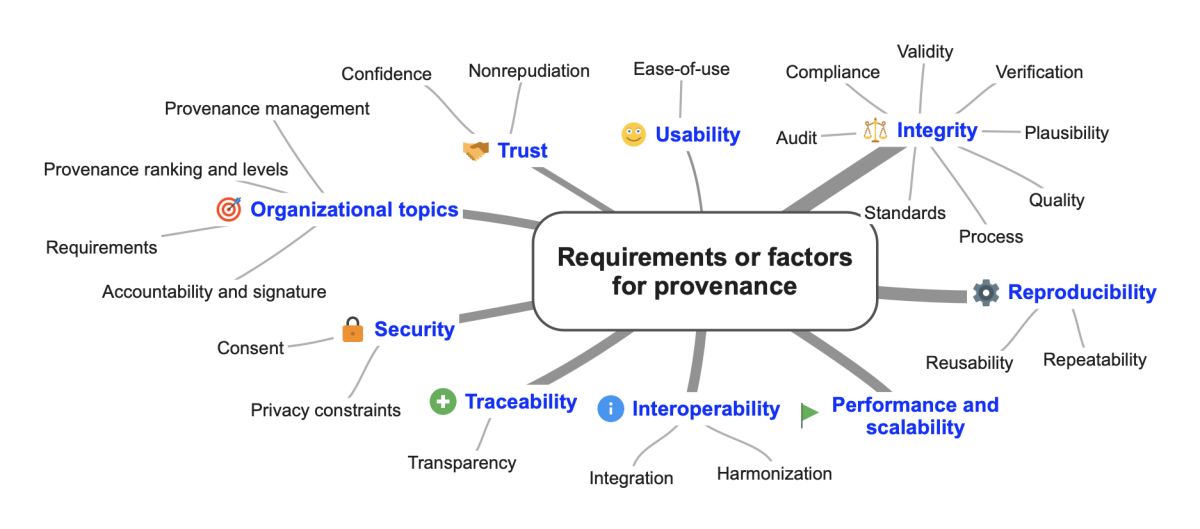
Reported provenance requirements or factors by word fields. The line thickness in the first level proportionally reflects the respective characteristics count. The second level displays all occurred requirement classes.

Out of 66 reviewed papers, 44 (67%) papers mentioned ≥1 functional and nonfunctional requirements for the referenced framework type. However, 33% (22/66) of the papers did not identify any specific requirements. For those studies that did, we identified 9 different word fields, matched them (Figure 3), and explained the citations (Multimedia Appendix 6).

Intensive interdisciplinary work on requirements analysis has been undertaken [75,83]. As such, a workshop with scientists from multiple disciplines (biology, computer science, ecology, and chemistry) and an exploratory study [75] contribute to requirements collection in the epidemiological field [83]. Another way to identify requirements is based on exhaustive literature research and interviews with domain specialists [85]. Figure 3 visualizes the reported provenance requirements. The most popular requirements refer to the word fields integrity (16/44, 36%) and reproducibility (13/44, 30%), followed by interoperability, traceability, and performance or scalability related topics (each 9/44, 20%). Others were related to the word fields organizational and security (each 8/44, 18%). Only a few studies reported on trust (5/44, 11%) and usability (3/44, 7%) linked approaches.

#### RQ1.6: Domain-Specific Conditions Including Guidelines

Some papers reported domain-specific standards for provenance (n=17). Beyond the W3C standards, we identified the Open Archival Information System Functional Model as a basis for the development of a research object concept [32]. Another example is the International Organization for Standardization (ISO) 15489-1, which defines the term *metadata* [51] or the Technical Committee 276 “Biotechnology” ISO, which standardizes provenance information for the biotechnology domain [74]. The National Institutes of Health guideline “Rigor and Reproducibility” [49] addresses topics impeding the study replicability. The FAIR principles explicitly guided data management practices in studies [77,83]. The FAIRSCAPE framework presented standards and interoperability such as JSON-LD, W3C PROV or CAT, DOI, and EVI’s formal model as extension to W3C as evidence chains that support or challenge a result [81].

### RQ2: Potential Value of Provenance Information

#### RQ2.1: Impact of Provenance Information

**Figure 4:**
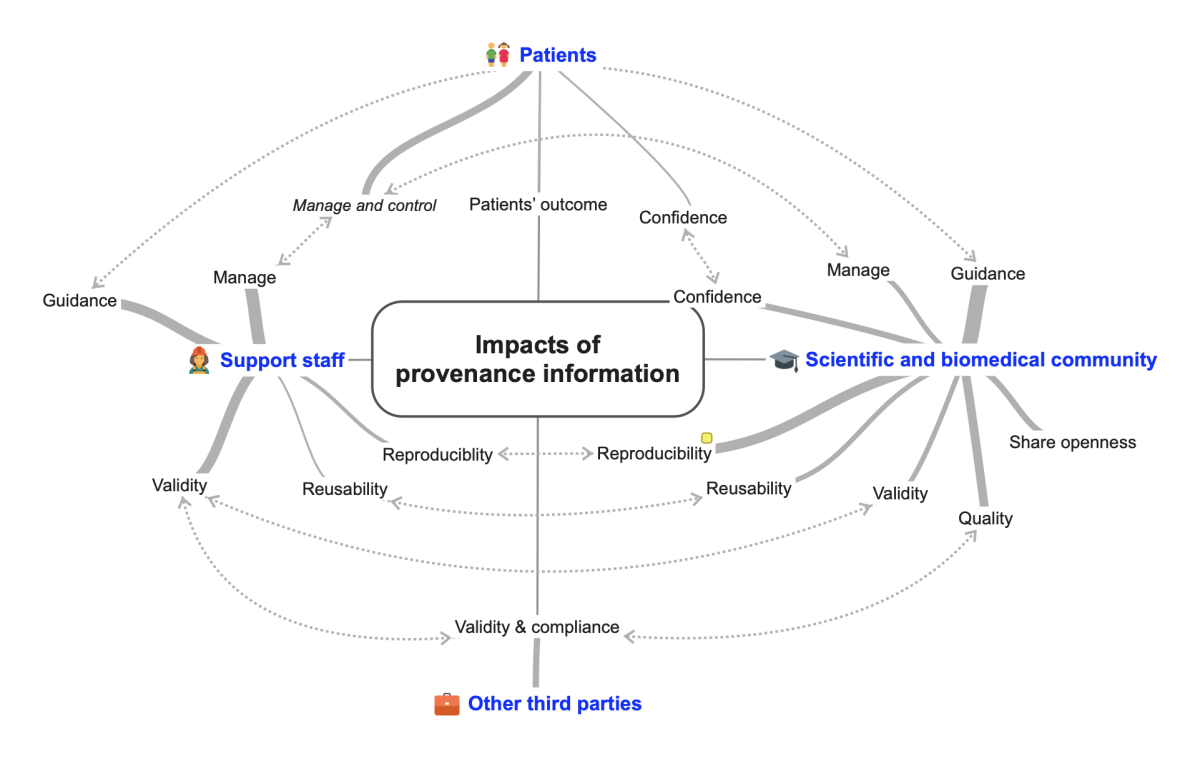
Reported impacts of provenance information. Level 1 presents the stakeholder groups and level 2 presents the impacts on the stakeholders. The line thickness in the second level proportionally reflects the respective counts of the characteristics.

In our review, a total of 42 papers reported about various impacts (n=99) on different stakeholders (Multimedia Appendix 7). The availability of provenance data impacts the scientific and biomedical communities (Figure 4). With regard to the work of researchers, scientists, academia, investigators, and clinicians (n=64), most papers reported guidance benefits (19/64, 30%) and reproducibility-related effects (12/64, 19%). Considerably fewer papers observed validity (4/64, 6%), managing influence (4/64, 6%), reusability (5/64, 8%) and confidence effects (6/64, 9%). Other studies reported that provenance information impacts the willingness to share knowledge (6/64, 9%), for example, by providing a unified repository for the experimental data for the research group [82,85]. Interestingly, only 13% (8/64) of the studies discussed implications on the quality of research (eg, [46,49,52,72,77,78,84]).

Other involved team or staff members (n=22) such as developers, data managers, or domain experts were also affected by the availability of provenance information. The majority recognizes benefits in validity (5/22, 23%) [26,40,49,60,61] and managing benefits (8/22, 36%) [27,33,41,56,71,76,80,83] followed by guidance benefits (5/22, 23%) [33,53,60,67,83]. In addition, reproducibility (3/22, 14%) [26,60,79] and reusability (1/22, 4%) impacts were mentioned.

Only low impact on patients (n=7) was described, mostly referring to the consent of their data (5/7, 71%) [26,45,48,56,69] to an improved measurable patient outcome and trust in evidence for clinical recommendations (each 1/7, 14%) [57].

Only a few effects on other third parties (n=6) such as data privacy officers, authorities, government, or industry were reported. Related implications concerned mainly the evidence for data validity or sensitive data-processing solutions [26,45,48,56,69,83].

#### RQ2.2: Data Sources

A large number of papers (n=42) reported studies processed different types of data sources to generate provenance information, for example, neurological data [1,28,84,85], with large-scale data from the brain imaging component of the UK Biobank project [82], electronic health record data [27], study data [62], animals data [80], pathology data [74], omics data [52], biomedical or medical data [64,77,78], computational data [72], time-series analysis of neonatal intensive care unit data [81], and data from hybrid methods [69].

### RQ3: Challenges, Problems, and Bottlenecks

**Figure 5:**
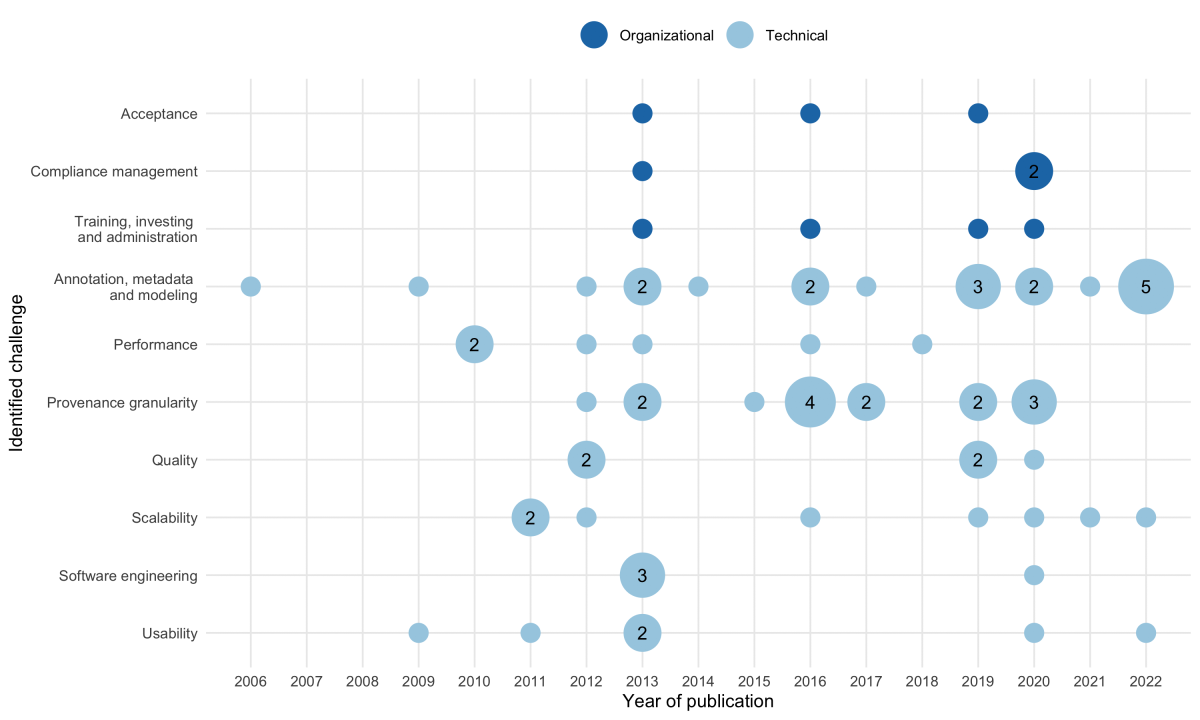
Challenges per year of publication. The size of and the numbers in the circles represent the number of articles that reveal a challenge (color-coded). Note that numbers are omitted for single articles per category.

Overall, 47 papers reported 74 distinct challenges impeding the implementation of provenance. We categorized these challenges into organizational and technical groups, provided details (Multimedia Appendix 8) and presented a temporal overview of the challenges (Figure 5). In summary, issues are related to data annotation, metadata, and modeling of provenance, as well as performance-related challenges. However, the need for more detailed provenance information; consideration of compliance managing topics (eg, security-related conditions); and adherence to quality and software engineering principles such as exchange, discovery, and interoperability emerged later in the course. Furthermore, usability and scalability questions emerged very early in context with provenance consumption.

More than three-quarters of the reported challenges are technical (64/74, 86%). Thereof, approximately one-fourth is associated with provenance granularity issues (15/64, 23%). Curcin et al [1] pointed out that a granular tracking of relevant human interactions, automated processes, or logging is needed and emphasized the difficulty of choosing a proper level of granularity of provenance and associated with this, the right semantic complexity [4,57]. Beyond that, a balanced trade-off between fast execution and provenance granularity must be found [42]. In fact, a fine-granular provenance level impacts the computing and storage resources [57,58]. Furthermore, managing sensitive data restriction requires the integration of adequate security level granularity into the provenance model [56].

Approximately one-third of the reported challenges (20/64, 31%) either mention the insufficient availability of metadata—which subsequently leads to incomplete provenance models—that claims the terminological heterogeneity in the metadata terms associated with study data sets [84] or does not conform to explicit annotation standards [82]. An improved availability of provenance metadata and FAIR enrichment of the data was demanded [29,38,80]. Furthermore, stakeholders should be involved in the semantic enrichment of provenance data [4,51]. However, during this metadata annotation phase, a lack of semiautomated procedures for ontology selection, semantic modeling, or mapping techniques was reported [2,29,51]. Although the use of existing models is encouraged [59], as it improves semantic interoperability [29], the reuse of vocabularies to represent provenance information remains an extensive task [29]. Cheng et al [70] noted that it was necessary to properly integrate domain-specific demands into the provenance model. Ruiz-Olazar et al [85] claimed that a unified data model for handling metadata is still missing.

One-fifth of the studies (14/64, 22%) reported performance problems during the acquisition of provenance data, such as workflow overhead [54,73] and scalability [13,85] issues. Increasing data volumes hamper the processing of provenance visualization and stream handling [76]. One proposal with respect to the cost-intensive visualization was to reduce the size of large provenance graphs [39]. Other authors reported challenges with quality [26,29,36,41,52] and usability [54,64,69,70,73,75]. According to the literature, data quality and reuse are lacking due to the deficit in provenance deployment, particularly for observational and administrative studies [26]. Furthermore, the lack of information about experimental origins in genomics data and their related systematic quality control assessment reduce the quality of provenance and the level of creditability [52]. In particular, the low uptake of high-quality semantic models [9] and the unavailability of provenance in general [36] cause information loss and data quality issues. A minor concern is the usability because provenance is recognized to be still in infancy [73]. The challenge of applying more software engineering techniques (4/64, 6%) [4,32,42,67] was reported to facilitate provenance implementation across a broad range of software tools in the biomedical domain and beyond [1].

Significantly fewer organizational challenges (10/74, 13%) [1,4,38,56-58,64,69,73] were reported, partly attributable to a basic unawareness of provenance benefits and less exchange between stakeholders. Khan et al [58] stressed that provenance capture must be established as a standard practice, not as an afterthought. McClatchey et al [64] also recommended working toward gaining the stakeholder’s acceptance and confidence in the infrastructure. In the same vein, it is recommended to integrate developers already in the design phase [1]. However, financial challenges were reported due to the necessary investments in provenance-enabled tooling and capabilities [4]. The upcoming relevance of patient-mediated data handling raised new challenges and requirements, especially with respect to policy and governance topics [69] and rigorous validation approaches [74].

### RQ4: Demands

Because of the extensive information obtained from RQ1, we extended the RQs to gain more insights into the provenance tracing and classification requirements identified in RQ1.

Interestingly, most of the 15 papers referred to claims relating to quality aspects.

For example, a more robust assessment of data quality is required [36], and clearer and more consistent policies and policy ontologies are requested to prevent disclosure of sensitive data [56] and more trained staff is required [50], including data managers, software architects, or semantic web specialists. User-friendly interfaces should help scientists in the provenance querying process [54]. Developers should not only recognize technologies but also data model recommendations during the design phase [1]. Performance of provenance reasoning needs to be improved [68] and the further development of ontologies needs to be automated [4,49]. The term “intelligible machines” rather than “intelligent machines” was suggested to better respect the specific aspects of big data technologies in medical research [57]. Integrating the Healthcare Enterprise standards, health care legacy protocols, interoperability, and legacy issues are furthermore mentioned [48], and mappings between entities of various provenance models should be completed [65]. Future integration into a recognized ISO standard similar to BioCompute was proposed [60].

### RQ5: Completeness of Provenance Information

The literature predominantly reports on a qualitative evaluation of completeness during the data management processes. However, we found one study describing a data management process dealing with metadata for traceability in clinical studies, which delivered complete provenance in this respect [12]. Curcin [4] proposed to incorporate provenance information in the validation against standards.

One study applied data from 6 clinical research studies and >100 variables to evaluate the coverage of the provenance ontology in the semantic annotation of the study descriptions [49]. Two other documents invoked the need for minimal information elements to ensure sufficient process specification [28] and the existence of rich provenance information for reconstructing and rerunning pipelines [29].

A visualization of provenance data in neuroimaging took a semiqualitative approach for measuring the coverage. They mapped the metrics to use cases for the traceability of results and concluded that there is no absolute measure possible to verify the visualization approach [61]. Arshad et al [61] tested 15% of their workflows for verifiability of results, comparability of workflows, progression of the data for the analysis and origin of results, and evolution to see how data products evolved during an experiment.

Furthermore, Sahoo et al [38] examined the proportion of provenance metadata information across research articles using a qualitative hypothesis method. The method also provides a provenance ranking algorithm for the computation of a reproducibility rank for each article. The outcome of the self-contained DataLad data set presented valid, machine-actionable provenance information for every single result file of the performed data processing [82].

Numerical indication of completeness was not achieved in any of the other papers. However, the papers pointed out the advantages of provenance capture, for example, related to the longevity and accessibility of data after years [40].

### Road Map for a Tailor-Made Provenance Framework

**Figure 6:**
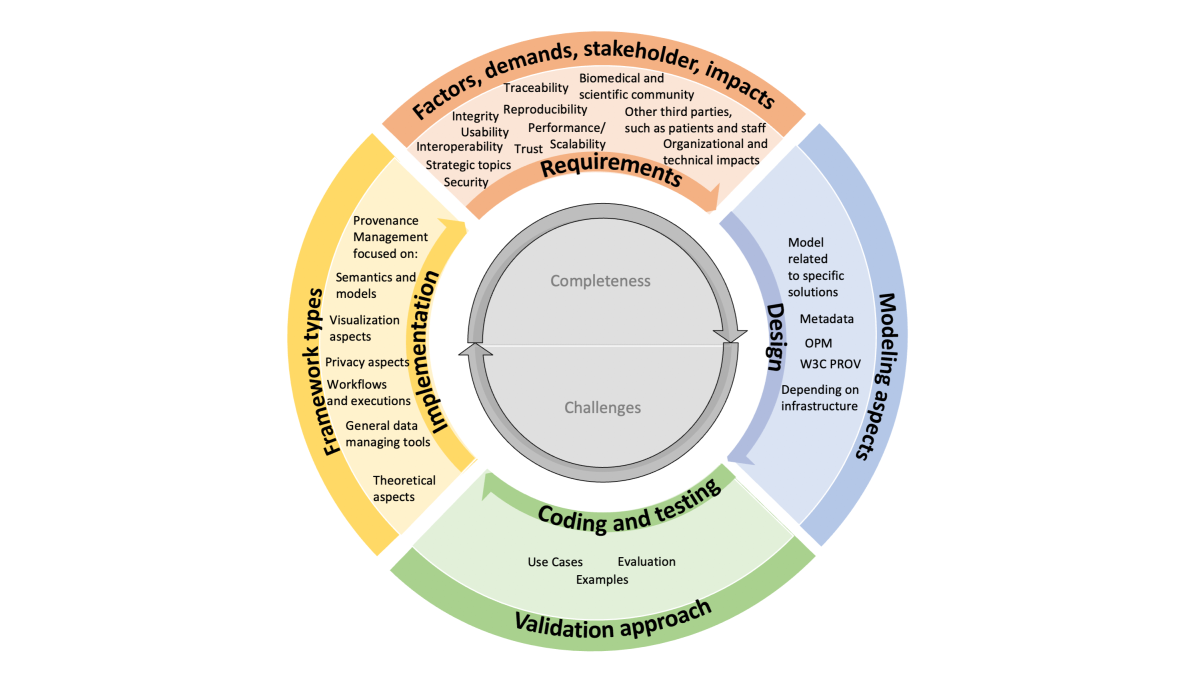
Road map toward a tailor-made provenance framework (Provenance-Software Framework Lifecycle [SFL]). The road map shows the 4 major processing phases in the inner circle segments: starting with the requirements definition, setup of the design based on the requirements, followed by coding and testing phases related to the given requirements and the implementation after successful testing. The outer and innermost circle present the mapped sections from our research questions approach to the Provenance-SFL. OPM: Open Provenance Model; W3C: World Wide Web Consortium.

On the basis of the insights obtained from the literature review, we developed a road map for the implementation of a tailor-made provenance framework (Provenance-SFL). This approach is based on the SFL for the development, provision, and management of software [87]. The heterogeneous tracking approaches, their artifacts, and varying degrees of fulfillment of the RQs are depicted in Figure 6 and determine our main discussion points.

## Discussion

### Principal Findings

This scoping review presents various approaches and criteria for provenance tracking as a crucial aspect of research, especially in the biomedical domain. Our holistic view leads to an extensive summary of pooled research results, provides possible answers to the 5 RQs and discloses current knowledge gaps.

Following the previously published scoping review protocol and the described deviation led us to include 66 full-text papers from initially 764 papers found in the PubMed and Web of Science databases. Using a structured and pretested data extraction sheet, contextual, but detailed, results were extracted to answer the 5 RQs defined in the protocol. The results of the scoping review led us to propose a Provenance-SFL road map, which distinguishes between the framework types and model characteristics, the validation status, and the requirement and provenance characteristics (Figure 6).

Most models in this review referred to the W3C PROV and OPM standards. As shown in Figure 2, an increased number of papers relate to the implementation frameworks published between 2016 and 2022. One reason for the increase in implementations might be the substantiation to extend W3C PROV and OPM [58]. Another reason might be the increasing awareness of data management practices. At present, heterogeneous data sources, dynamic infrastructures, data exchange across boundaries, and a lack of standards for quality measures characterize the state of electronic health record data sets [48].

A provenance framework must acknowledge the semantic complexity of the domain and its relevant facets and requirements [58] and the importance of good data management tooling and practices (Figure 2). In addition to requirements analysis, a thorough strategy is necessary to plan the typical data management steps such as collecting, managing, and analyzing data [30]. According to Curcin [4], validation readiness can be achieved by separating modeling and verification of provenance data from the software implementation. We agree that precise requirements analysis, as part of the software life cycle, and the subsequent individual life cycle steps, such as testing and maintenance procedures, support the consequent temporal evolution and hence improve the quality of provenance frameworks and applications.

When incorporated in an inspection, provenance information must be sufficient for a content-related validation against applicable and accepted standards [4]. Therefore, precise validation methods for provenance services regarding usability and performance, scalability, fault tolerance, and functionality are needed [64]. We saw that validation approaches are linked to the evolution of provenance modeling and subsequent implementation attempts. Curcin et al [1] argued that it was necessary to launch more formal software engineering techniques to foster provenance implementation across a broad range of software tools in the biomedical domain and beyond. In that sense, formal validation as part of the software engineering process contributes to increased software and data quality. Formal validation requires testing efforts and testing evidence. Accurate alignment of testing procedures against predefined requirements in the software life cycle could not be identified in the included papers.

Provenance information is of high value for the scientific and biomedical community (eg, researchers); support staff (eg, developers); patients and other third parties (eg, data privacy officer or authority; Figure 4). It is interesting to see that despite the high impact of provenance (Multimedia Appendix 7), only some stakeholders provide sufficient provenance information. Rather, it appears that responsibility for overall provenance management is being shifted to the support staff [88]. We argue that available technology, IT knowledge, and data management skills need to be paired with both domain-specific knowledge and combined with constraints of legal nature or guidance [4,50]. This complexity indeed results in a very time-consuming business. However, automation and metadata collection can support this process [4,6]. As a matter of fact, good provenance information strengthens the credibility of the data and proves that data have not been intentionally or unintentionally changed throughout the data life cycle [89].

We believe that the persisting disagreement on the interpretation of the term *provenance* hampers the uptake of existing frameworks. A unique understanding of the concepts surrounding provenance should be developed, followed by engineering efforts for modeling, implementation, and validation interventions. The ISO 8000-2:2022 standard [90] defines the term *data quality* and clearly recommends defining degrees of requirements. This definition should be considered for use in provenance systems.

With regard to the implementation of provenance systems, we observed that increasing legal and scientific demands require research projects to be implemented more transparently. However, the granularity of provenance could not yet be resolved and so-called knowledge bottlenecks [50,65] persist. It is important to understand that appropriate modeling of provenance information and effective provenance management techniques are required to protect sensitive provenance data.

It furthermore remains unclear how to scale provenance systems for high amounts of data [2,58] (eg, how to store and represent provenance information in an aggregated and efficient manner or how to assist users in sophisticated provenance queries [13]). Without doubt, automated and scalable solutions become impelling due to new challenges arising from the disposal and use of permanently increasing computing power [40]. Growing focus is on the usability of the interface, particularly when provenance systems are implemented in the broad medical community, including patients, physicians, and researchers [73].

The lack of mandatory specifications or guidelines for provenance capture might be the reason why other papers only mention partial completeness. We strongly recommend doing more research on completeness checks as part of provenance tracing. The level of completeness and accuracy of provenance information of core data elements, especially in real-world data, could reveal data integrity issues and thus, affect the overall validity of the study results. Furthermore, reproducibility significantly depends on the accuracy of provenance information. For example, Mondelli et al [27] delivered a tool for better scientific and longitudinal data management, which supports users, reproducibility by provenance, and reproduction through docker containers.

Interestingly, the concept of “quality of provenance” is not clearly defined in any of the papers included for this review. We believe that data quality issues need to be addressed to reach completeness, accuracy, and timeliness of the data and to create trust in it.

Another direction reveals the importance of good and systematic data management practices [51] and the coordination with relevant stakeholders through the data life cycle. As such, first approaches toward interdisciplinary collaboration started within the MIRAPIE community project [91] and were also recognized in the CAESAR project [75].

In summary, our review collects and structures the challenges during the accomplishment of provenance capture (Figure 5). Challenges that address missing or lacking organizational and technical capabilities were triangulated into more specific subcategories such as organizational (eg, investment and training and administrative) and technical (eg, granularity, performance and modeling and metadata annotation, delimitation reproducibility, and replicability) challenges.

### Comparison With Prior Work

In the realm of similar studies, a recent systematic literature review by Sembay et al [92] delved into provenance data management in health information systems. This review is a valuable resource if interested in the technical aspects, encompassing various methods, models, methodologies, and technologies associated with provenance data management in health information systems. It followed the guidelines proposed by Kitchenham and Charters [93] for performing systematic literature reviews in software engineering. Another systematic literature review by Ahmed et al [94] studied the impacts of data provenance in health care and General Data Protection Regulation compliance–based data provenance from a technological perspective. Although our RQ1 shares conceptual and domain-related similarities with the compared studies, there are notable differences in scope, RQs, methodological approaches, and thematic analysis focus.

More than a year after we published our protocol and concluded the thematic analysis of our review findings, a scoping review on biomedical data provenance was published [95]. The authors acknowledged and referenced our protocol. As they had anticipated, our review comprised 5 broad RQs, with the primary overlap occurring in RQ1. However, our scoping review offers more comprehensive results concerning the practical application of provenance and the associated challenges, including aspects such as completeness and validation. In addition, we provide in-depth descriptions of thematic areas and their subtopics, supported by tables, figures, and, most notably, a development of a tailor-made provenance framework road map. These aspects can be considered as the added value of our findings.

Our scoping review shares only few references with the other 2 (in detail: 11 out of 66 references are identical with references used in Johns et al [95] and 2 references are identical with those used in Sembay et al [92]). Therefore, all 4 reviews examine different research results and focus on other aspects. Ahmed et al [94] did not provide the list of the selected 59 articles.

### Strengths and Limitations

This work applied a rigorous scoping review methodology using the framework developed by Arksey and O’Malley [16]. All screening stages were carried out by at least 2 independent reviews of 4 members. A previously published protocol [17] guided our review. The fact that the scoping review includes comprehensive results for the 5 related RQs and a road map for a tailor-made Provenance-SFL framework with many additional results as supplements can be considered a strength of this review. The criteria for categorization of provenance impact, requirements, and challenge classes were identified qualitatively in peer review.

One limitation may be because we excluded gray literature from our analysis.

### Conclusions

In this paper, we highlighted various approaches and criteria for provenance tracking together with their referenced artifacts, and we developed a road map for a tailor-made Provenance-SFL framework.

Provenance capture benefits all stakeholders involved in data processing, but it is associated with manifold and individual challenges during design, implementation, and the active use scenario phase.

Sophisticated data management planning, documentation, metadata expression, and automation along the sensitive data-processing pipelines need to be scrutinized and implemented throughout the data life cycle and in adherence to the underlying infrastructure condition. With rising data volumes and the legal and scientific demands, there is an urgent need for greater transparency in implementing provenance systems in research projects, despite the challenges of unresolved granularity and knowledge bottlenecks. In addition, the roles and responsibilities of a data stewardship escorting the data should be expressed in this context and intensive training and education measures should be put in place. Guidance and recommendations are requested to provide the systematic measurement of provenance and calls for defining a minimal or gold standard. Governance for good data management and scale-up of good data management capabilities matter in this regard.

The mentioned artifacts, particularly those related to quality aspects, can be seen as transition points resulting from incomplete preliminary work. Therefore, harmonized engineering efforts are now necessary to overcoming the existing hurdles. Awareness of these challenges can facilitate an easier qualified and accurate provenance construction and auditable consumption while enforcing FAIR principles and interoperability standards for data sharing. The effect of provenance for data quality monitoring and the impact of expressive metadata on provenance quality can be considered as open RQs for future work.

## Appendix

Multimedia Appendix 1 Search strings and queries used for database search.

Multimedia Appendix 2 PRISMA-ScR (Preferred Reporting Items for Systematic Reviews and Meta-Analyses extension for Scoping Reviews) checklist. Filled-in checklist items for the scoping review.

Multimedia Appendix 3 Document characteristics of the study corpus. Document characteristics of the study corpus containing presentation of studies between 2006 and 2022 and allocation to the target domain.

Multimedia Appendix 4 Studies and their respective assignment to a framework type. Framework types are displayed by categories and characteristics of provenance. “Framework—practical provenance management” comprises practical efforts for development and implementation of a provenance approach. “Framework—theoretical provenance management” approach includes ideas and generic principles for provenance consideration.

Multimedia Appendix 5 Included articles and their related model. Included articles grouped by their related model respectively by using similar approaches. Counts for categories and subcategories are given per model group and approach.

Multimedia Appendix 6 Reported requirements or factors for provenance. Studies cited to classification, description, and counting of requirement or factor terms.

Multimedia Appendix 7 Reported impacts and stakeholders of provenance information. Included articles and counting of impacts per category; shows the structure and relationship between the individual stakeholders and the reported impacts. The comprehensive meaning of the impact is explained in the column “Description” by the assignment of the individual statements from the mentioned papers.

Multimedia Appendix 8 Reported technical and organizational challenges. Included articles and counting per category and subcategory for reported challenges, problems, and bottlenecks during accomplishment of provenance.

## Abbreviations

FAIR: findable, accessible, interoperable, reusable
ISO: International Organization for Standardization
OPM: Open Provenance Model
PRISMA: Preferred Reporting Items for Systematic Reviews and Meta-Analyses
PRISMA-ScR: Preferred Reporting Items for Systematic Reviews and Meta-Analyses Extension for Scoping Reviews
RQ: research question
SFL: Software Framework Life Cycle
W3C: World Wide Web Consortium

## Data Availability

All data generated or analyzed during this study are included in this published manuscript and its supplementary information files.

## References

[1] Curcin V, Miles S, Danger R, Chen Y, Bache R, Taweel A (2014). Implementing interoperable provenance in biomedical research. Future Gener Comput Syst.

[2] Jayapandian CP, Zhao M, Ewing RM, Zhang GQ, Sahoo SS (2012). A semantic proteomics dashboard (SemPoD) for data management in translational research. BMC Syst Biol.

[3] Cuggia M, Combes S (2019). The French health data hub and the German medical informatics initiatives: two national projects to promote data sharing in healthcare. Yearb Med Inform.

[4] Curcin V (2017). Embedding data provenance into the learning health system to facilitate reproducible research. Learn Health Syst.

[5] Wodke JA, Michaelis L, Henkel R (2023). The MeDaX knowledge graph prototype. Stud Health Technol Inform.

[6] Schröder M, Staehlke S, Groth P, Nebe JB, Spors S, Krüger F (2022). Structure-based knowledge acquisition from electronic lab notebooks for research data provenance documentation. J Biomed Semantics.

[7] Johnson KE, Kamineni A, Fuller S, Olmstead D, Wernli KJ (2014). How the provenance of electronic health record data matters for research: a case example using system mapping. EGEMS (Wash DC).

[8] PROV-overview. World Wide Web Consortium.

[9] Wilkinson MD, Dumontier M, Aalbersberg IJ, Appleton G, Axton M, Baak A, Blomberg N, Boiten J, da Silva Santos LB, Bourne PE, Bouwman J, Brookes AJ, Clark T, Crosas M, Dillo I, Dumon O, Edmunds S, Evelo CT, Finkers R, Gonzalez-Beltran A, Gray AJ, Groth P, Goble C, Grethe JS, Heringa J, 't Hoen PA, Hooft R, Kuhn T, Kok R, Kok J, Lusher SJ, Martone ME, Mons A, Packer AL, Persson B, Rocca-Serra P, Roos M, van Schaik R, Sansone S, Schultes E, Sengstag T, Slater T, Strawn G, Swertz MA, Thompson M, van der Lei J, van Mulligen E, Velterop J, Waagmeester A, Wittenburg P, Wolstencroft K, Zhao J, Mons B (2016). The FAIR Guiding Principles for scientific data management and stewardship. Sci Data.

[10] Inau ET, Sack J, Waltemath D, Zeleke AA (2021). Initiatives, concepts, and implementation practices of FAIR (findable, accessible, interoperable, and reusable) data principles in health data stewardship practice: protocol for a scoping review. JMIR Res Protoc.

[11] Jauer ML, Deserno TM (2020). Data provenance standards and recommendations for FAIR data. Stud Health Technol Inform.

[12] Hume S, Sarnikar S, Noteboom C (2020). Enhancing traceability in clinical research data through a metadata framework. Methods Inf Med.

[13] Sahoo SS, Nguyen V, Bodenreider O, Parikh P, Minning T, Sheth AP (2011). A unified framework for managing provenance information in translational research. BMC Bioinformatics.

[14] Yakutovich AV, Eimre K, Schütt O, Talirz L, Adorf CS, Andersen CW, Ditler E, Du D, Passerone D, Smit B, Marzari N, Pizzi G, Pignedoli CA (2021). AiiDAlab – an ecosystem for developing, executing, and sharing scientific workflows. Comput Mater Sci.

[15] Schissel D, Abla G, Flanagan SM, Greenwald M, Lee X, Romosan A, Shoshani A, Stillerman J, Wright J (2014). Automated metadata, provenance cataloging and navigable interfaces: ensuring the usefulness of extreme-scale data. Fusion Eng Des.

[16] Arksey H, O'Malley L (2005). Scoping studies: towards a methodological framework. Int J Soc Res Methodol.

[17] Gierend K, Krüger F, Waltemath D, Fünfgeld M, Ganslandt T, Zeleke AA (2021). Approaches and criteria for provenance in biomedical data sets and workflows: protocol for a scoping review. JMIR Res Protoc.

[18] Braun V, Clarke V (2006). Using thematic analysis in psychology. Qual Res Psychol.

[19] Ouzzani M, Hammady H, Fedorowicz Z, Elmagarmid A (2016). Rayyan-a web and mobile app for systematic reviews. Syst Rev.

[20] Van Rossum G, Drake FL (2009). Python 3 Reference Manual.

[21] R Core Team The R project for statistical computing. R Foundation for Statistical Computing.

[22] Wickham H, Averick M, Bryan J, Chang W, McGowan L, François R, Grolemund G, Hayes A, Henry L, Hester J, Kuhn M, Pedersen T, Miller E, Bache S, Müller K, Ooms J, Robinson D, Seidel D, Spinu V, Takahashi K, Vaughan D, Wilke C, Woo K, Yutani H (2019). Welcome to the Tidyverse. J Open Source Softw.

[23] Tricco AC, Lillie E, Zarin W, O'Brien KK, Colquhoun H, Levac D, Moher D, Peters MD, Horsley T, Weeks L, Hempel S, Akl EA, Chang C, McGowan J, Stewart L, Hartling L, Aldcroft A, Wilson MG, Garritty C, Lewin S, Godfrey CM, Macdonald MT, Langlois EV, Soares-Weiser K, Moriarty J, Clifford T, Tunçalp Ö, Straus SE (2018). PRISMA extension for scoping reviews (PRISMA-ScR): checklist and explanation. Ann Intern Med.

[24] Page MJ, McKenzie JE, Bossuyt PM, Boutron I, Hoffmann TC, Mulrow CD, Shamseer L, Tetzlaff JM, Akl EA, Brennan SE, Chou R, Glanville J, Grimshaw JM, Hróbjartsson A, Lalu MM, Li T, Loder EW, Mayo-Wilson E, McDonald S, McGuinness LA, Stewart LA, Thomas J, Tricco AC, Welch VA, Whiting P, Moher D (2021). The PRISMA 2020 statement: an updated guideline for reporting systematic reviews. BMJ.

[25] Daumke P, Heitmann KU, Heckmann S, Martínez-Costa C, Schulz S (2019). Clinical text mining on FHIR. Stud Health Technol Inform.

[26] Parciak M, Bauer C, Bender T, Lodahl R, Schreiweis B, Tute E, Sax U (2019). Provenance solutions for medical research in heterogeneous IT-infrastructure: an implementation roadmap. Stud Health Technol Inform.

[27] Mondelli ML, Magalhães T, Loss G, Wilde M, Foster I, Mattoso M, Katz D, Barbosa H, de Vasconcelos AT, Ocaña K, Gadelha LM (2018). BioWorkbench: a high-performance framework for managing and analyzing bioinformatics experiments. PeerJ.

[28] Keator DB, Helmer K, Steffener J, Turner J, Van Erp T, Gadde S, Ashish N, Burns G, Nichols B (2013). Towards structured sharing of raw and derived neuroimaging data across existing resources. Neuroimage.

[29] Celebi R, Rebelo Moreira J, Hassan AA, Ayyar S, Ridder L, Kuhn T, Dumontier M (2020). Towards FAIR protocols and workflows: the OpenPREDICT use case. PeerJ Comput Sci.

[30] Pimentel JF, Freire J, Murta L, Braganholo V (2019). A survey on collecting, managing, and analyzing provenance from scripts. ACM Comput Surv.

[31] Bánáti A, Kacsuk P, Kozlovszky M (2017). Reproducibility analysis of scientific workflows. Acta polytech Hung.

[32] Bechhofer S, Buchan I, De Roure D, Missier P, Ainsworth J, Bhagat J, Couch P, Cruickshank D, Delderfield M, Dunlop I, Gamble M, Michaelides D, Owen S, Newman D, Sufi S, Goble C (2013). Why linked data is not enough for scientists. Future Gener Comput Syst.

[33] Groth P, Moreau L (2011). Representing distributed systems using the open provenance model. Future Gener Comput Syst.

[34] Golbeck J, Hendler J (2008). A Semantic web approach to the provenance challenge. Concurr Comput.

[35] Schuchardt KL, Gibson T, Stephan E, Chin G (2008). Applying content management to automated provenance capture. Concurr Comput.

[36] Danese MD, Halperin M, Duryea J, Duryea R (2019). The generalized data model for clinical research. BMC Med Inform Decis Mak.

[37] Tyndall T, Tyndall A (2018). FHIR healthcare directories: adopting shared interfaces to achieve interoperable medical device data integration. Stud Health Technol Inform.

[38] Sahoo SS, Valdez J, Kim M, Rueschman M, Redline S (2019). ProvCaRe: characterizing scientific reproducibility of biomedical research studies using semantic provenance metadata. Int J Med Inform.

[39] Xu S, Rogers T, Fairweather E, Glenn A, Curran J, Curcin V (2018). Application of data provenance in healthcare analytics software: information visualisation of user activities. AMIA Jt Summits Transl Sci Proc.

[40] Huber SP, Zoupanos S, Uhrin M, Talirz L, Kahle L, Häuselmann R, Gresch D, Müller T, Yakutovich AV, Andersen CW, Ramirez FF, Adorf CS, Gargiulo F, Kumbhar S, Passaro E, Johnston C, Merkys A, Cepellotti A, Mounet N, Marzari N, Kozinsky B, Pizzi G (2020). AiiDA 1.0, a scalable computational infrastructure for automated reproducible workflows and data provenance. Sci Data.

[41] Madougou S, Santcroos M, Benabdelkader A, van Schaik BD, Shahand S, Korkhov V, van Kampen AH, Olabarriaga SD (2012). Provenance for distributed biomedical workflow execution. Stud Health Technol Inform.

[42] Guedes T, Martins LB, Falci ML, Silva V, Ocaña KA, Mattoso M, Bedo M, de Oliveira D (2020). Capturing and analyzing provenance from spark-based scientific workflows with SAMbA-RaP. Future Gener Comput Syst.

[43] Thavasimani P, Cala J, Missier P (2019). Why-diff: exploiting provenance to understand outcome differences from non-identical reproduced workflows. IEEE Access.

[44] Marinho A, de Oliveira D, Ogasawara E, Silva V, Ocaña K, Murta L, Braganholo V, Mattoso M (2017). Deriving scientific workflows from algebraic experiment lines: a practical approach. Future Gener Comput Syst.

[45] Danger R, Curcin V, Missier P, Bryans J (2015). Access control and view generation for provenance graphs. Future Gener Comput Syst.

[46] Moreau L (2011). Provenance-based reproducibility in the Semantic Web. J Web Semant.

[47] Holland DA, Seltzer MI, Braun U, Muniswamy‐Reddy KK (2008). PASSing the provenance challenge. Concurr Comput.

[48] Margheri A, Masi M, Miladi A, Sassone V, Rosenzweig J (2020). Decentralised provenance for healthcare data. Int J Med Inform.

[49] Sahoo SS, Valdez J, Rueschman M (2016). Scientific reproducibility in biomedical research: provenance metadata ontology for semantic annotation of study description. AMIA Annu Symp Proc.

[50] Curcin V, Soljak M, Majeed A (2013). Managing and exploiting routinely collected NHS data for research. Inform Prim Care.

[51] Razick S, Močnik R, Thomas LF, Ryeng E, Drabløs F, Sætrom P (2014). The eGenVar data management system--cataloguing and sharing sensitive data and metadata for the life sciences. Database (Oxford).

[52] Saccone SF, Quan J, Jones PL (2012). BioQ: tracing experimental origins in public genomic databases using a novel data provenance model. Bioinformatics.

[53] Woodman S, Hiden H, Watson P (2017). Applications of provenance in performance prediction and data storage optimisation. Future Gener Comput Syst.

[54] Marinho A, Murta L, Werner C, Braganholo V, Cruz SM, Ogasawara E, Mattoso M (2012). ProvManager: a provenance management system for scientific workflows. Concurr Comput.

[55] Bowers S, McPhillips TM, Ludäscher B (2008). Provenance in collection‐oriented scientific workflows. Concurr Comput.

[56] Can O, Yilmazer D (2020). A novel approach to provenance management for privacy preservation. J Inf Sci.

[57] Curcin V, Fairweather E, Danger R, Corrigan D (2017). Templates as a method for implementing data provenance in decision support systems. J Biomed Inform.

[58] Khan FZ, Soiland-Reyes S, Sinnott RO, Lonie A, Goble C, Crusoe MR (2019). Sharing interoperable workflow provenance: a review of best practices and their practical application in CWLProv. Gigascience.

[59] Ciccarese P, Soiland-Reyes S, Belhajjame K, Gray AJ, Goble C, Clark T (2013). PAV ontology: provenance, authoring and versioning. J Biomed Semantics.

[60] Alterovitz G, Dean D, Goble C, Crusoe MR, Soiland-Reyes S, Bell A, Hayes A, Suresh A, Purkayastha A, King CH, Taylor D, Johanson E, Thompson EE, Donaldson E, Morizono H, Tsang H, Vora JK, Goecks J, Yao J, Almeida JS, Keeney J, Addepalli K, Krampis K, Smith KM, Guo L, Walderhaug M, Schito M, Ezewudo M, Guimera N, Walsh P, Kahsay R, Gottipati S, Rodwell TC, Bloom T, Lai Y, Simonyan V, Mazumder R (2018). Enabling precision medicine via standard communication of HTS provenance, analysis, and results. PLoS Biol.

[61] Arshad B, Munir K, McClatchey R, Shamdasani J, Khan Z (2019). NeuroProv: Provenance data visualisation for neuroimaging analyses. J Comput Lang.

[62] Stitz H, Luger S, Streit M, Gehlenborg N (2016). AVOCADO: visualization of workflow-derived data provenance for reproducible biomedical research. Comput Graph Forum.

[63] Sahoo SS, Bodenreider O, Hitzler P, Sheth A, Thirunarayan K (2010). Provenance context entity (PaCE): scalable provenance tracking for scientific RDF data. Proceedings of the 22nd International Conference on Scientific and Statistical Database Management.

[64] McClatchey R, Branson A, Anjum A, Bloodsworth P, Habib I, Munir K, Shamdasani J, Soomro K, neuGRID Consortium (2013). Providing traceability for neuroimaging analyses. Int J Med Inform.

[65] Monnin P, Legrand J, Husson G, Ringot P, Tchechmedjiev A, Jonquet C, Napoli A, Coulet A (2019). PGxO and PGxLOD: a reconciliation of pharmacogenomic knowledge of various provenances, enabling further comparison. BMC Bioinformatics.

[66] Ornelas T, Braga R, David JM, Campos F, Castro G (2018). Provenance data discovery through semantic web resources. Concurr Comput.

[67] Madougou S, Shahand S, Santcroos M, van Schaik B, Benabdelkader A, van Kampen A, Olabarriaga S (2013). Characterizing workflow-based activity on a production e-infrastructure using provenance data. Future Gener Comput Syst.

[68] Lim C, Lu S, Chebotko A, Fotouhi F (2011). Storing, reasoning, and querying OPM-compliant scientific workflow provenance using relational databases. Future Gener Comput Syst.

[69] Jennath HS, Anoop VS, Asharaf S (2020). Blockchain for healthcare: securing patient data and enabling trusted artificial intelligence. Int J Interact Multimed Artif Intell.

[70] Cheng X, Pizarro R, Tong Y, Zoltick B, Luo Q, Weinberger DR, Mattay VS (2009). Bio-swarm-pipeline: a light-weight, extensible batch processing system for efficient biomedical data processing. Front Neuroinform.

[71] Jabal AA, Bertino E (2018). A comprehensive query language for provenance information. Int J Coop Info Syst.

[72] Kim J, Deelman E, Gil Y, Mehta G, Ratnakar V (2008). Provenance trails in the Wings/Pegasus system. Concurr Comput.

[73] Lim C, Lu S, Chebotko A, Fotouhi F, Kashlev A (2013). OPQL: querying scientific workflow provenance at the graph level. Data Knowl Eng.

[74] Wittner R, Mascia C, Gallo M, Frexia F, Müller H, Plass M, Geiger J, Holub P (2022). Lightweight distributed provenance model for complex real-world environments. Sci Data.

[75] Samuel S, König-Ries B (2022). A collaborative semantic-based provenance management platform for reproducibility. PeerJ Comput Sci.

[76] Yazici IM, Aktas MS, Yazici IM, Aktas MS (2022). A novel visualization approach for data provenance. Concurr Comput.

[77] Ross PB, Song J, Tsao PS, Pan C (2021). Trellis for efficient data and task management in the VA Million Veteran program. Sci Rep.

[78] Nadendla S, Jackson R, Munro J, Quaglia F, Mészáros B, Olley D, Hobbs ET, Goralski SM, Chibucos M, Mungall CJ, Tosatto SC, Erill I, Giglio MG (2022). ECO: the evidence and conclusion ontology, an update for 2022. Nucleic Acids Res.

[79] Westbrook A, Varki E, Thomas WK (2021). RepeatFS: a file system providing reproducibility through provenance and automation. Bioinformatics.

[80] Raboudi A, Allanic M, Balvay D, Hervé PY, Viel T, Yoganathan T, Certain A, Hilbey J, Charlet J, Durupt A, Boutinaud P, Eynard B, Tavitian B (2022). The BMS-LM ontology for biomedical data reporting throughout the lifecycle of a research study: from data model to ontology. J Biomed Inform.

[81] Levinson MA, Niestroy J, Al Manir S, Fairchild K, Lake DE, Moorman JR, Clark T (2021). FAIRSCAPE: a framework for FAIR and reproducible biomedical analytics. Neuroinformatics.

[82] Wagner AS, Waite LK, Wierzba M, Hoffstaedter F, Waite AQ, Poldrack B, Eickhoff SB, Hanke M (2022). FAIRly big: a framework for computationally reproducible processing of large-scale data. Sci Data.

[83] Mitchell SN, Lahiff A, Cummings N, Hollocombe J, Boskamp B, Field R, Reddyhoff D, Zarebski K, Wilson A, Viola B, Burke M, Archibald B, Bessell P, Blackwell R, Boden LA, Brett A, Brett S, Dundas R, Enright J, Gonzalez-Beltran AN, Harris C, Hinder I, David Hughes C, Knight M, Mano V, McMonagle C, Mellor D, Mohr S, Marion G, Matthews L, McKendrick IJ, Mark Pooley C, Porphyre T, Reeves A, Townsend E, Turner R, Walton J, Reeve R (2022). FAIR data pipeline: provenance-driven data management for traceable scientific workflows. Philos Trans A Math Phys Eng Sci.

[84] Wang X, Wang Y, Ambite JL, Appaji A, Lander H, Moore SM, Rajasekar AK, Turner JA, Turner MD, Wang L, Sahoo SS (2022). Enabling scientific reproducibility through FAIR data management: an ontology-driven deep learning approach in the NeuroBridge project. AMIA Annu Symp Proc.

[85] Ruiz-Olazar M, Rocha ES, Vargas CD, Braghetto KR (2021). The neuroscience experiments system (NES)-a software tool to manage experimental data and its provenance. Front Neuroinform.

[86] Moreau L, Freire J, Futrelle J, McGrath R, Myers J, Paulson P (2008). The open provenance model: an overview. Proceedings of the 2nd International Provenance and Annotation Workshop on Provenance and Annotation of Data and Processes.

[87] Morrison P, Moye D, Pandita R, Williams L (2018). Mapping the field of software life cycle security metrics. Inf Softw Technol.

[88] Gierend K, Freiesleben S, Kadioglu D, Siegel F, Ganslandt T, Waltemath D (2023). The status of data management practices across German medical data integration centers: mixed methods study. J Med Internet Res.

[89] Wing JM (2019). The data life cycle. Harv Data Sci Rev.

[90] ISO 8000-2:2022 data quality. International Organization for Standardization.

[91] Gierend K, Wodke JA, Genehr S, Gött R, Henkel R, Krüger F, Mandalka M, Michaelis L, Scheuerlein A, Schröder M, Zeleke A, Waltemath D (2023). TAPP: defining standard provenance information for clinical research data and workflows - obstacles and opportunities. Proceedings of the 2023 ACM Web Conference.

[92] Sembay MJ, de Macedo DD, Júnior LP, Braga RM, Sarasa-Cabezuelo A (2023). Provenance data management in health information systems: a systematic literature review. J Pers Med.

[93] Kitchenham B, Charters S (2007). Guidelines for performing systematic literature reviews in software engineering. School of Computer Science and Mathematics.

[94] Ahmed M, Dar AR, Helfert M, Khan A, Kim J (2023). Data provenance in healthcare: approaches, challenges, and future directions. Sensors (Basel).

[95] Johns M, Meurers T, Wirth FN, Haber AC, Müller A, Halilovic M, Balzer F, Prasser F (2023). Data provenance in biomedical research: scoping review. J Med Internet Res.

