# Supplementary material for: Provenance Information for Biomedical Data and Workflows: Scoping Review
Source: J Med Internet Res. 2024 Aug 23;26:e51297. doi: 10.2196/51297 (PMC11380065; doi:10.2196/51297)
Supplement: Multimedia Appendix 1 [file jmir_v26i1e51297_app1.docx]

**Multimedia Appendix 1. Search strings**. Queries used for database search.

**Pubmed** [1]

("biomed*"[Title/Abstract] OR "Biomedical Research"[MeSH Terms] OR "EHR"[Title/Abstract] OR "healthcare"[Title/Abstract] OR "electronic health record"[Title/Abstract] OR "Electronic Health Records"[MeSH Terms] OR "clinical"[Title/Abstract] OR "scientific"[Title/Abstract]) AND ("provenance"[Title/Abstract] OR "prov"[Title/Abstract] OR "lineage"[Title/Abstract]) AND ("interoperab*"[Title/Abstract] OR "data"[Title/Abstract] OR "metadata"[Title/Abstract] OR Metadata[Mesh] OR"workflow*"[Title/Abstract] OR "Workflow"[MeSH Terms] OR "semantic"[Title/Abstract] OR "framework"[Title/Abstract] OR "annotat*"[Title/Abstract] OR "ontolog*"[Title/Abstract] OR "management"[Title/Abstract] OR "document*"[Title/Abstract] OR Documentation[Mesh] OR "model"[Title/Abstract]) AND (("audit*"[Title/Abstract] OR "decision support"[Title/Abstract] OR "Decision Support Techniques"[MeSH Terms] OR "ETL"[Title/Abstract] OR "Extract-Transform-Load"[Title/Abstract] OR "FHIR"[Title/Abstract] OR "record linking"[Title/Abstract] OR "Medical Record Linkage"[MeSH Terms] OR "machine learning"[Title/Abstract] OR "reproducib*"[Title/Abstract] OR "transparen*"[Title/Abstract] OR "track*"[Title/Abstract]OR "implement*"[Title/Abstract]) NOT "cell"[Title/Abstract])

**Web of Science** [2]

**(TS=biomed* or TS=EHR or TS=healthcare or TS="Electronic Health Record" or TS=clinical or TS=scientific ) AND**

**(TS=provenance or TS=prov or TS=lineage) AND**

(TS=interoperab* or TS=(data NEAR/2 (flow or quality or transformation) ) or TS=metadata or TS=workflow or TS=semantic or TS=framework or TS=annotat* or TS=ontolog* or TS=management or
TS=document* or TS=(model NEAR/2 provenance)) AND

**(TS=audit* or TS='decision support' or TS='ETL' or TS="Extract-Transform-Load" or TS=FHIR or TS='record linking' or TS='machine learning' or TS=reproducib* or TS=transparen* or TS=track* or TS=implement*)**

References

[1] Website: NIH National Library of Medicine. Available from: https://pubmed.ncbi.nlm.nih.gov/ [accessed Mar23, 2021].

[2] Website: Web of Science^TM^. Available from: https://clarivate.com/login/ [accessed Mar23, 2021].
