# Supplementary material for: Provenance Information for Biomedical Data and Workflows: Scoping Review
Source: J Med Internet Res. 2024 Aug 23;26:e51297. doi: 10.2196/51297 (PMC11380065; doi:10.2196/51297)

**Multimedia Appendix 3. Document characteristics of the study corpus.** Document characteristics of the study corpus containing presentation of studies between 2006 and 2022 and allocation to the target domain.

|  | Document characteristics | Count | Reference^a^ |
| --- | --- | --- | --- |
|  |  |  |  |
| **Year of publication** |  |  |  |
|  | 2006 - 2008 | 5 | 12,13,26,35,53 |
|  | 2009 - 2011 | 6 | 5,11,25,43,49,51 |
|  | 2012 - 2014 | 13 | 4,9,10,20,30,31,32,34,39, 44,45,48,54 |
|  | 2015 - 2017 | 9 | 8,17,18,23,24,29,33,37,42 |
|  | 2018 - 2020 | 21 | 1,2,3,6,7,14,15,16,19,21,22,27,28,36,38,40,41,46,47,50,52 |
|  | 2021 - 2022 | 12 | 55,56,57,58,59,60,61,62,63,64,65,66 |
| **Target domain** |  |  |  |
|  | (Bio-) medical or healthcare domain | 45 | 1,2,3,4,5,6,9,10,13,14,15,16,17,18,20,24,27,28,29,30,31,32,34,36,37,38,39,40,41,42,43,44,45,46,50,51,58,59,60,61,62,63,64,65,66 |
|  | Domain independent | 21 | 7,8,11,12,19,21,22,23,25,26,33,35,47,48,49,52,53,54,55,56,57 |

^a^Number corresponds to column “SNo” in Table 1, main document


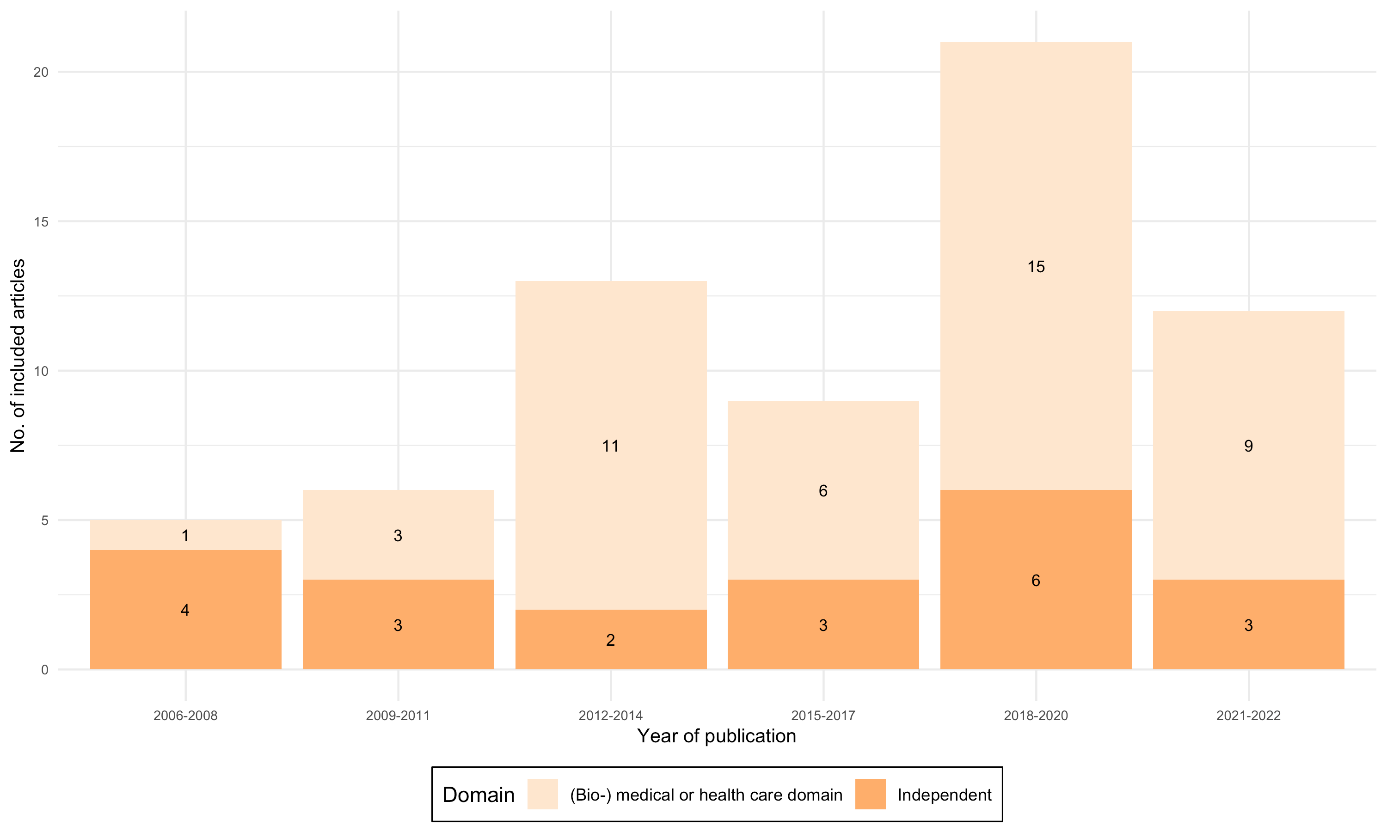

Supplement: Multimedia Appendix 3 [file jmir_v26i1e51297_app3.docx]
