# Supplementary material for: Provenance Information for Biomedical Data and Workflows: Scoping Review
Source: J Med Internet Res. 2024 Aug 23;26:e51297. doi: 10.2196/51297 (PMC11380065; doi:10.2196/51297)

**Multimedia Appendix 4. Studies and their respective assignment to a framework type**. Studies and their respective assignment to a framework type. Framework types are displayed by categories and characteristics of provenance. “Framework—practical provenance management” comprises practical efforts for development and implementation of a provenance approach. “Framework—theoretical provenance management” approach includes ideas and generic principles for provenance consideration.

| Category | Subcategory | Count | Reference^c^ |
| --- | --- | --- | --- |
|  | |  |  |
| Framework type – Practical Provenance^a^ | | 58 |  |
|  | Semantics & models, ontologies & metadata | 27 | 4,5,8,10,11,14,16,25,26,28,29,31,32,35,37,39,43,44,46,47,49,51,52,55,59,61,65 |
|  | Scientific workflows and workflow execution | 18 | 3,6,12,13,19,20,21,23,33,34,38,45,48,53,54,58,60,63 |
|  | Privacy aspect | 5 | 15,24,27,36,50 |
|  | Visualization aspect | 4 | 18,41,42,57 |
|  | General data managing tools | 4 | 56,62,64,66 |
| Framework type– Theoretical Provenance^b^ | | 8 |  |
|  | Different reviews, recommendations, or approaches from initiatives | 8 | 1,2,7,9,17,22,30,40 |

**^a^**Comprised development of a provenance-related solution with focus on given constraints

**^b^**Included ideas or principles on which a provenance frame is based (rather than with practice and experiment)

^c^Number corresponds to column “SNo” in Table 1, main document


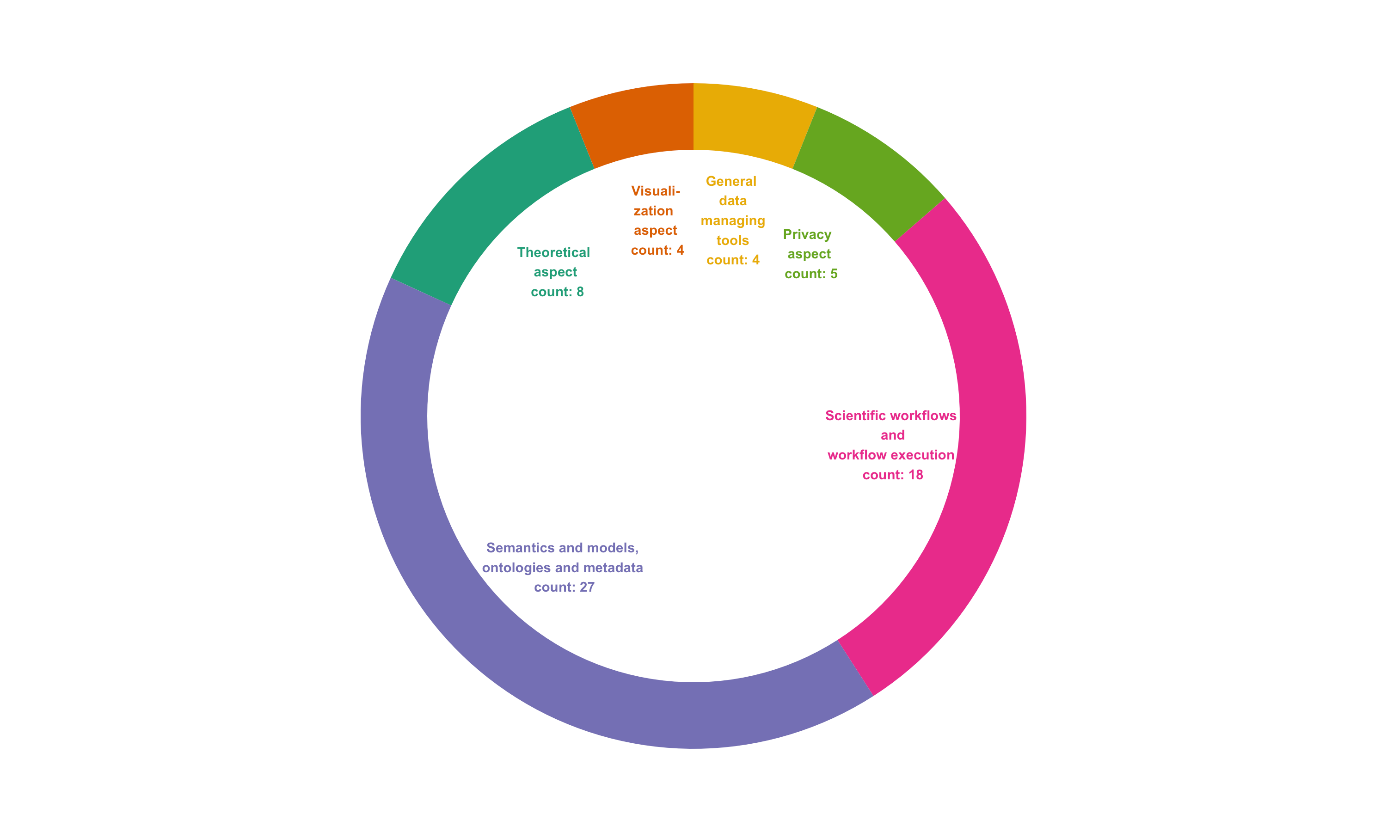

Supplement: Multimedia Appendix 4 [file jmir_v26i1e51297_app4.docx]
