# Supplementary material for: Provenance Information for Biomedical Data and Workflows: Scoping Review
Source: J Med Internet Res. 2024 Aug 23;26:e51297. doi: 10.2196/51297 (PMC11380065; doi:10.2196/51297)
Supplement: Multimedia Appendix 5 [file jmir_v26i1e51297_app5.docx]

**Multimedia Appendix 5. Included articles and their related model**. Included articles grouped by their related model respectively by using similar approaches. Counts for categories and subcategories are given per model group and/or approach.

| Category | Subcategory | Count | Reference^e^ |
| --- | --- | --- | --- |
|  |  |  |  |
| W3C PROV^a^ |  | 25 |  |
|  | FHIR | 1 | 15 |
|  | W3C PROV extension | 7 | 16,21,23,29,39,62,65 |
|  | W3C PROV-* | 12 | 1,2,22,27,37,38,40,41,55,57,61,64 |
|  | W3C PROV-* and other | 4 | 4,6,46,56 |
|  | W3C, Dublin Core, Research Object | 1 | 10 |
| OPM^b^ |  | 17 |  |
|  | OPM | 13 | 3,5,11,20,24,25,33,34,36,43,45,47,48 |
|  | OPM extension | 2 | 49,54 |
|  | OPM and other | 2 | 9,52 |
| Model related to specific solutions^c^ |  | 11 |  |
|  | ADES model | 1 | 19 |
|  | BERT | 1 | 32 |
|  | CDISC ODM | 1 | 28 |
|  | COMAD | 1 | 35 |
|  | CRIM | 1 | 17 |
|  | Mathematical model | 1 | 8 |
|  | Provenance data model for an AI/ML model | 1 | 50 |
|  | RDBM model | 2 | 14,51 |
|  | Data model based on Bento Graph Model Description Framework | 1 | 58 |
|  | NES data model | 1 | 66 |
| Metadata^d^ |  | 5 |  |
|  | Semantic model using metadata | 5 | 12,13,30,53,59 |

^a^Conceptual data model from W3C; ^b^Model transforming process; ^c^Different models applied for specific requirements; ^d^Building a semantic model based on metadata; ^e^Number corresponds to column “SNo” in Table 1, main document; *Placeholder for different PROV model extensions.
