# Supplementary material for: Provenance Information for Biomedical Data and Workflows: Scoping Review
Source: J Med Internet Res. 2024 Aug 23;26:e51297. doi: 10.2196/51297 (PMC11380065; doi:10.2196/51297)
Supplement: Multimedia Appendix 6 [file jmir_v26i1e51297_app6.docx]

**Multimedia Appendix 6. Reported requirements or factors for provenance.** Studies cited to classification, description, and counting of requirement or factor terms.

|  | Mapped terms | Description | Count | Reference ^a^ |
| --- | --- | --- | --- | --- |
|  |  |  |  |  |
| **Integrity** | Plausibility, Validity, Quality, Audit, Process, Compliance, Standard, Verification | Kind of evidence that given procedures are true and accurate and in adherence to demands or guidelines | 16 | 9,12,15,18,25,27,29,32,33,41,43,47,53,55,57,62 |
| **Inter-opera-bility** | Harmonization, Integration, Interoperability | Ability of programs/machines/humans to exchange information | 9 | 4,11,27,38,50,52,55,61,64 |
| **Organi-zational topics** | Management, Ranking, Requirements, Signature, Accountability, Information, Level | Need of setting user requirements, ranking provenance, accountability at levels of research organizations for increased usage of provenance, strategic management of provenance | 8 | 2,6,15,16, 30,33,47,52 |
| **Perfor-mance and scalability** | Performance and scalability | Perspective execution times and storage, orchestration depending on how-to-compute and what-to-compute | 9 | 9,27,51,52,54 56,58,63,66 |
| **Repro-ducibility** | Reproducibility, Reusability, Repeatability | Ability that a result can be produced or done again in the same way; repeating experiments | 13 | 6,8,12,16,19,22,29,33,37,42,47,49,56 |
| **Security** | Privacy, Consent | Considering privacy aware aspects, but allow access to metadata | 8 | 9,24,27,36,50,52 ,58,64 |
| **Trace-ability** | Transparency, Traceability | Operations on data are easy to understand and operations are trackable | 9 | 16,18,28,30,40,50,62,64,65 |
| **Usability** |  | ease-of-use (and re-use) incentive for researchers | 3 | 56,62,64 |
| **Trust** | Trust, Confidence, Non-repudiation | Believe in and be sure about the abilities on processing of data | 5 | 18,25,29,43,55 |

^a^ Number corresponds to column “SNo” in Table 1, main document
