# Supplementary material for: Provenance Information for Biomedical Data and Workflows: Scoping Review
Source: J Med Internet Res. 2024 Aug 23;26:e51297. doi: 10.2196/51297 (PMC11380065; doi:10.2196/51297)
Supplement: Multimedia Appendix 7 [file jmir_v26i1e51297_app7.docx]

**Multimedia Appendix 7. Reported impacts and stakeholders of provenance information.** Included articles and counting of impacts per category; shows the structure and relationship between the individual stakeholders and the reported impacts. The comprehensive meaning of the impact is explained in the column “Description” by the assignment of the individual statements from the mentioned papers.

| Category | Description | Count | Reference^a^ |
| --- | --- | --- | --- |
|  |  |  |  |
| **Scientific and biomedical community** |  | **64** |  |
| Confidence | - FHIR supports provenance assertions of data as a critical foundation for authenticity and trust - trustworthiness of data is crucial for their produced data - confidence in results due to reproducibility - trust in evidence for clinical recommendations - support reliable and shareable computational research results. - increase trust in well-founded scientific conclusions | 6 | 1,11,25,37,62,64 |
| Managing influence | - crucial for assigning responsibility for decisions - meet GDPR requirements by implementing data provenance in directory services - meet privacy preservation requirements - platform for managing healthcare data | 4 | 11,15,36,50 |
| Openness | - make scientific workflows more open by FAIRification of the involved datasets and applying semantic technology - enhance exchange and improve security and usability throughout the health data continuum - efficient sharing of all artifacts resulting from a workflow - support future reuse - open about researchers work, better assess scientific evidence by inspecting its provenance - association of process provenance with captured FAIR 1.2 outcomes | 6 | 6,15,38,62, 64,63 |
| Quality | - ensure quality of results by reproducibility - provenance metadata to ensure quality and validate quality of data - origin and quality control process are traceable - improvement of execution performance by working on optimized transformations - improve the pace and quality of scientific research in large biomedical studies - reduces risk of user error - demonstrates a potential for interpretable search results and a potential for iterative boosting - Consistent capture of evidence information with ECO allows tracking of annotation provenance, establishment of quality control measures, and evidencebased data mining. | 8 | 25,29,32,53, 58,59,60,65 |
| Reproducibility | - FHIR supports provenance assertions of data as a critical foundation for reproducibility - investigate critical dependencies in scientific workflows - reproducibility based on scientific data publication model and on notion of research objects - facilitating reproducible research by automatically capturing the trace of the research task - making computational science fully reproducible, in compliance with FAIR principles - contributes to reproducibility - provenance as a program with executable semantics for reproducing results - scientific reproducibility - enables standardized reporting of data provenance by clearing bottleneck for downstream analysis - productivity (due to parallel data processing capabilities) and efficiency (time saving for researchers), facilitates replication of results and decreases storage space requirements - end-to-end provenance management from the beginning of an experiment to its end - promotes reproducibility | 12 | 1,8,10,17,19,21,25,29,40,51, 56,60 |
| Reusability | - produces human-readable explanations of the impact of workflow differences on observed output differences - reuse of methods for reruns such as BDBag approach based on BagIt which supports large-scale workflow data - even a non-expert person will be able to understand the general meaning of the local term - domain-agnostic framework which provides standard interfaces, comprehensive process provenance capture for computational reproducibility - providing a unified repository for the experimental data of an entire research group | 5 | 22,38,61,63,66 |
| Guidance | - guide to help scientists to select tools that best address their specific problems - allows users to access study data and provenance metadata - making computational science more user-friendly - dynamic reports for analyzing provenance data of scientific workflows at runtime - description of experiment and tracking of provenance - enable decision making process and patient consent auditing - querying on data - allow scientists to focus on the essence of their experiments - organized and annotate resources, useable for archiving - the BioComputeObject supports standardized reporting of genomic sequence data provenance - identifying differences by visualization - provenance service to facilitate improved clinical decisions making - increase knowledge: answer competency questions towards complete framework - enhance scientist's knowledge about an experiment - figure out potential sources of workflow errors - free users from low-level details of provenance storage, querying a simple query language, intuitive results as a graph - fitness-for-purpose analysis - End-users do not need to learn a new programming language to use services provided by FAIRSCAPE - enables researchers to efficiently perform the management of their experimental data in a secure and user-friendly environment | 19 | 7,16,19,21,23,27,31,34,39,40,42,45,46,47,48,54, 55, 62,66 |
| Validity | - allows clear data provenance to researchers - safe and valid provenance graphs allowing maximum amount of information based on security model - to ensure accuracy and validity - visualized provenance for verification of result or an intermediate result | 4 | 14,24,40,41 |
| **Support staff** | developer, bioinformatician, system administrator, domain expert, data manager, support | **22** |  |
| Manage | - workflow systems coupled with execution provenance tracking and analysis enables better scientific data management - troubleshooting problems, improving performance - allows post-mortem workflow execution analysis - detect privacy breaches - secure, interoperable and multigranular provenance framework - to look up a file in a data registry and see what (if any) metadata is held on it, and what other versions of that data product also exist - collection of local terms and their mapping to other levels should be done by a referent person (ontologist, data manager) interacting with the research team - benefits with regard to analysis and troubleshooting | 8 | 3,11,20, 36,52, 57,61,64 |
| Reproducibility | - assist in answering domain-specific and domain- agnostic questions regarding the provenance of data sets - encourage interoperability and the reproducibility of bioinformatics protocols - RepeatFS reduces the risk of user error and promotes reproducibility | 3 | 2,40,60 |
| Reusability | guides developers through high-level, reusable recommendations | 1 | 9 |
| Guidance | - troubleshooting problems, assigning responsibility for decisions, improving performance - operational auditing for medical datasets - allow easy user interaction and creation of BCOs - figure out workflow errors - during FAIR management of data and models | 5 | 11,33,40,48,64 |
| Validity | - transparency - making computational science more transparent - unambiguousness of data - ensure accuracy and validity - visualized provenance for verification of result or an intermediate result | 5 | 2,19,29,40,41 |
| **Patients** |  | **7** |  |
| Confidence | trust in evidence for clinical recommendations | 1 | 37 |
| Manage and control | - seek informational value from provenance records - access regulation to EHR and provenance data - patients consent auditing - manage and trace the accessibility of their medical records - enables patients to be the true owner of their data | 5 | 2,24,27,36,50 |
| Patients’ outcome | measurable positive impact on practitioner performance that leads to directly attributable and measurable improvement in patient outcomes | 1 | 37 |
| **Other 3rd parties** | data privacy officer, authority, government, auditor, industry | **6** |  |
| Validity | - transparency for data privacy officer - make security solution understandable and verifiable - incentive agreements with the third parties for the data sharing, etc. - tracing of complex health documents - clarify the results from clinical trials and other genomic related data for regulatory submissions - support policymakers in openly justifying their decision | 6 | 2,24,27,40,50,64 |

^a^Number corresponds to column “SNo” in Table 1, main document
