# Supplementary material for: Provenance Information for Biomedical Data and Workflows: Scoping Review
Source: J Med Internet Res. 2024 Aug 23;26:e51297. doi: 10.2196/51297 (PMC11380065; doi:10.2196/51297)
Supplement: Multimedia Appendix 8 [file jmir_v26i1e51297_app8.docx]

**Multimedia Appendix 8. Reported technical and organizational challenges, problems, and bottlenecks.** Included articles and counting per category and subcategory for reported challenges, problems, and bottlenecks during accomplishment of provenance.

|  | Subcategory | Description | Count | Reference^a^ |
| --- | --- | --- | --- | --- |
|  |  |  |  |  |
| **Organi-zational** |  |  | **10** |  |
|  | Acceptance | Avoidance of big data technologies rather than understanding the key aspects for medical research to build research |  | 37 |
|  |  | Accept provenance capture as a standard practice rather as an afterthought and follow-up on best practices |  | 38 |
|  |  | Gain stakeholder’s acceptance and confidence in infrastructure |  | 45 |
|  | Training & Investing,  Administration | Involve Developers need not only to know which technologies to use, but what principles to follow in their designs |  | 9 |
|  |  | Lack of address of social and administrative challenges by biomedical research community |  | 16 |
|  |  | Major investment for provenance-enabled tooling and capabilities of provenance analytics |  | 17 |
|  |  | Scale-up of PoC with limited data management capabilities to incorporate new stakeholders and other entities |  | 50 |
|  | Compliance Management | Integration of policy ontologies into the proposed model to prevent disclosure of sensitive data |  | 36 |
|  |  | implementing new security and privacy protocols for data sharing, authentication, interoperability, confidentiality requirements, electronically verifiable consents, and policy enforcement and governance for patient mediated data handling |  | 50 |
|  |  | To study provenance security, particularly compliance management |  | 54 |
| **Technical** |  |  | **64** |  |
|  | Provenance granularity |  | 15 |  |
|  |  | Practical tools for distributed access to neuroimaging data. Does not cover data access in terms of security. |  | 4 |
|  |  | Operating systems challenge on provenance storage due to the presence of the database on the operating system. |  | 7 |
|  |  | Obtainability of dependencies during execution due to incompleteness of data  (eg. unavailability of input data) |  | 8 |
|  |  | Focus on relevant provenance in processes |  | 9 |
|  |  | Deciding the level of granularity of provenance capture is a recognized problem in the field |  | 17 |
|  |  | Fidelity issue due to same presentation of provenance artifacts from system-level and user-generated events |  | 18 |
|  |  | Finding balanced trade-off between fast execution and provenance granularity to be addressed by domain experts |  | 21 |
|  |  | - Query effect of variation in workflow steps needs reconstruction of workflows and integration of "tacit knowledge" of involved scientists - Impossible to compare different workflows for the same experiment (eg. regarding execution time or variability in workflow steps) |  | 23 |
|  |  | Extend the OPM solution for provenance graphs to additional concepts introduced in PROV ontology or generic directed acyclic graphs. |  | 24 |
|  |  | To exploit PROV documents to enable reproducibility of clinical research |  | 27 |
|  |  | Little or no provenance captured during data analysis by the stakeholders in academia, industry, publisher |  | 30 |
|  |  | Lack of integration of policy ontology into the model and records causes disclosure of sensitive data |  | 36 |
|  |  | Add more templates that cover other scenarios, like non diagnostic decision support and implement more advanced PROV concepts such as hyperedges representing relations between more than two nodes |  | 37 |
|  |  | Lack of understandability of the shared resources |  | 38 |
|  | Annotation, Metadata and Modeling |  | 20 |  |
|  |  | Lack of provenance data limit the ability to query metadata across imaging resources without significant and time-consuming data mediation efforts |  | 4 |
|  |  | Incomplete human semantic modeling for making workflows FAIR need to be adapted by smart and intuitive workflow tools. Recommendation to work on prototype of FAIR workbench allows users to deal with workflows and protocols in a semantic and FAIR form. |  | 6 |
|  |  | 1. Generic model with limited provenance details for extension of model to domain specifics, usage of common terminologies |  | 9 |
|  |  | Lack of clear FAIR enrichment of data by researchers to improve the coverage and comprehensiveness of the ProvCaRe repository. |  | 16 |
|  |  | Provenance gap between the provenance metadata collected and the reporting requirements of different domains by stakeholders |  | 17 |
|  |  | Prior design limitation causes missing query possibility (from prov challenge) |  | 26 |
|  |  | Lack of extended metadata model to show data lineage and reproducibility within clinical research studies |  | 28 |
|  |  | Scarcity in automated approach to extend ontology for semantic annotation of research studies (use APIs) force to time consuming manual selection and importing of existing biomedical ontologies |  | 29 |
|  |  | Loosely defined constraints for adding metadata leads to incomplete entries and thereby reduces the quality of the database content. |  | 31 |
|  |  | Semantic complexity in provenance traces needs to satisfy domain constraints and user data requirements |  | 37 |
|  |  | Lack of dedicated constructs in provenance standards to represent the underlying hardware resource usage information as part of prospective or retrospective provenance. |  | 38 |
|  |  | Creating semi-automated mapping techniques, which can define initial mappings through use of lexical matching and subsequently reviewed by researchers to ensure accuracy. |  | 44 |
|  |  | High data maintenance effort in semantic web technologies to follow the evolution of associated databases |  | 46 |
|  |  | Proper integration of domain specific demands into the provenance model |  | 51 |
|  |  | Granularity challenge, as the two ontologies provide different granularities on terms, |  | 59 |
|  |  | BMS-LM is not detailed enough for the data, it includes not the biomedical researchers needs for flexible daily data annotation which is an issue for data public sharing because the local significance is not shared |  | 61 |
|  |  | Metadata used and produced by the framework does not conform to explicit annotation standard |  | 63 |
|  |  | including external resources and aggregating the metadata; |  | 64 |
|  |  | accessibility challenge to research data (discovery of relevant study data sets), terminological heterogeneity in the metadata terms associated with the study datasets |  | 65 |
|  |  | A unified data model for handling metadata is still an open research problem. The problem is compounded when the volume of collected data begins to grow. |  | 66 |
|  | Performance |  | 6 |  |
|  |  | Optimization of AEL derived workflow by doing algebraic transformations in order to reduce dataflow for performance |  | 23 |
|  |  | High overhead on workflow processing and high usage network bandwidth due to provenance gathering mechanism. |  | 34 |
|  |  | Reduce no. of RDF triples to increase performance of complex provenance queries |  | 43 |
|  |  | Investigate mechanism to improve the performance of provenance reasoning and leveraging techniques such as structural join algorithms and reachability query processing |  | 49 |
|  |  | Enhance performance of provenance queries by investigating index structures |  | 52 |
|  |  | To explore query optimization (study the performance of all 14 queries from the Third Provenance Challenge) |  | 54 |
|  | Quality |  | 5 |  |
|  |  | Deficiency in utilizing provenance to improve data quality and data reuse of observational and administrative data; ongoing extension of data sets and (federating) systems and of complexity of performing ETL makes the assessment of data quality a tedious task. |  | 2 |
|  |  | Deficiency in taking benefit from lessons learned by reusing high quality semantic models, but reproducibility issues occurred while reusing the models. |  | 6 |
|  |  | Unavailability of provenance causes Information loss and data quality assessment on data elements |  | 14 |
|  |  | Limitations mostly refer to the quality of the data that is possible to collect into the provenance store; semantic enrichment at all levels (data, processes, workflow descriptions and executions) to enable larger exploitation of the potential benefit of provenance data |  | 20 |
|  |  | Lack of source data and systematic quality control assessment; process flow diagrams should ideally include information on diagnostics and quality control (QC) |  | 32 |
|  | Scalability |  | 8 |  |
|  |  | Allow easy scalability in the number of triples for provenance as RDF reification |  | 5 |
|  |  | Better and more scalable Semantic Web reasoning techniques required to support the OPM specific inferences, and the necessary inferences required for reproducibility |  | 25 |
|  |  | Reduce big omics data size since this leads to spatial and temporal overheads |  | 38 |
|  |  | To reduce the size of large data provenance graphs with hundreds of nodes and design an effective combination of existing |  | 42 |
|  |  | Adaption to scalability of the system (SemPoD platform that uses semantic provenance) depending on the upgradation of the cache size of the server. |  | 44 |
|  |  | Major bottleneck of Blockchain to scalability, interoperability, and the delay in committing transactions. Improvement necessary to enable higher transaction throughput and better-managed services (data access smart devices) |  | 50 |
|  |  | As the data volume increases, provenance visualization and stream handling become difficult to store, process, and perform |  | 57 |
|  |  | Scalable storage for neuroimages |  | 66 |
|  |  |  |  |  |
|  | Software engineering |  | 4 |  |
|  |  | To rise to more formal software engineering techniques that will facilitate provenance implementation across a broad range of software tools in the biomedical domain and beyond. |  | 9 |
|  |  | Lack of metadata for reusability of Research Object in the context of linked data. |  | 10 |
|  |  | Adapting the SAMbA-RaP design to collect provenance from Data Frames as many applications are moving towards them |  | 21 |
|  |  | Extend the system with tools designed to gather information from log files. |  | 48 |
|  | Usability |  | 6 |  |
|  |  | Insufficient usability of user interface to help scientists in the provenance querying process |  | 34 |
|  |  | Insufficient usability of interface to Provenance Service  Help scientist in provenance querying process and guiding users by provenance query patterns. |  | 45 |
|  |  | Issues on integrating various aspects of the proposed system in terms of scalability, usability, accessibility and interoperability due the infancy stage of blockchain technology for enterprise applications. |  | 50 |
|  |  | Need for more user-friendly interfaces for wrapper management, storage management, task re-scheduling and traffic control, data source management. |  | 51 |
|  |  | Infancy state of usability research in provenance querying (while much research has been done on database usability) |  | 54 |
|  |  | small number of user participation for evaluation, mappings for the ontology-based data access required some manual curation |  | 56 |

^a^Number corresponds to column “SNo” in Table 1, main document
